# *Periplaneta americana* extract (L.) promotes hair regrowth in Alopecia areata mice by reducing inflammation and modulating skin microbiota

**DOI:** 10.3389/fphar.2025.1590648

**Published:** 2025-08-06

**Authors:** Tangfei Guan, Xin Yang, Canhui Hong, Jiali Zhu, Peiyun Xiao, Yongshou Yang, Chenggui Zhang, Zhengchun He

**Affiliations:** ^1^ Yunnan Provincial Key Laboratory of Entomological Biopharmaceutical R&D, College of Pharmacy, Dali University, Dali, China; ^2^ National-Local Joint Engineering Research Center of Entomoceutics, Dali, China; ^3^ West China School of Public Health, Sichuan University/West China Fourth Hospital, Chengdu, China

**Keywords:** Alopecia areata, *Periplaneta americana* (L.) extract, network pharmacology, transcriptomics, metabolomics, microbiomics

## Abstract

**Introduction:**

Alopecia areata (AA), an autoimmune hair-loss disease due to follicular inflammatory cell infiltration, lacks an ideal cure. The Periplaneta Americana (PA) extract (PA-011), a traditional Chinese medicine with anti-inflammatory, antioxidant, tissue-repair-promoting, and immune-regulating properties, was studied for its AA-treating effects.

**Methods:**

Guided by network pharmacology, AA model mice were treated with PA-011. Hair growth, skin tissue, inflammatory factors, and Wnt protein levels were examined. Transcriptomics, metabolomics, and 16S rRNA sequencing explored the hair-growth-promoting mechanisms.

**Results:**

Network pharmacology showed PA-011 could regulate AA-related targets and pathways. PA-011 intervention promoted hair follicle cell proliferation and hair growth in AA mice, reduced skin TNF-α, IL-23, and VCAM-1 expression. Transcriptomics and WB analysis indicated PA-011 downregulated inflammatory genes, activated Wnt3a, and modulated the TGF-β pathway. Metabolomics found PA-011 regulated metabolic pathways. 16S rRNA analysis showed it reversed AA-induced skin microbiota changes, inhibiting pathogens and promoting probiotics. Multi-omics analysis revealed PA-011 regulated skin microbiota and metabolic balance for hair growth.

**Discussion:**

In conclusion, PA-011 alleviates AA by reducing skin inflammation and modulating skin microbiota, suggesting its potential as an AA therapeutic.

## 1 Introduction

Alopecia areata (AA) is an autoimmune disease and the second most common non-scarring hair loss condition after androgenetic alopecia (He et al., 2024). The pathogenesis of AA is not fully understood, involving immune, genetic, psychological, and environmental factors (Dai et al., 2021). Sudden hair loss and disfigurement caused by AA impose psychological and economic burdens on patients, increasing the risk of poor mental health, low self-esteem, and psychiatric disorders (Dai et al., 2022). Pathogenic features of AA include hair follicle (HF) dystrophy, perifollicular lymphocyte infiltration, intense secretion of inflammatory cytokines, and lymphocyte-mediated attacks on anagen hair follicles (Maglakelidze et al., 2023). Currently, there are no definitive methods for preventing or treating AA, and most treatments have high relapse rates after discontinuation, often accompanied by side effects (Zheng et al., 2024).

Effective treatment options for AA are limited, with minoxidil (Freire et al., 2019), finasteride (Hosny et al., 2022), and JAK inhibitors being the primary options. Among these, JAK inhibitors are the only FDA-approved drugs for AA treatment (Wei et al., 2023). However, relapse or symptom exacerbation often occurs after discontinuation or dose reduction, and their long-term safety and immune tolerance effects remain unclear (Park et al., 2024), with a relapse rate as high as 50% (Younis et al., 2024). Minoxidil, as a first-line treatment for AA (Zhou et al., 2021), has a narrow indication range, noticeable side effects, and is not suitable for all types of hair loss (Moussa et al., 2022).

*Periplaneta americana* (L.) (PA) is one of the largest insects in the Periplaneta genus (Cao et al., 2024). In ancient Chinese medical texts such as Shennong Ben Cao Jing, its dried body is recorded as a traditional medicine (Zhao et al., 2024). Modern pharmacological studies have shown that *P. americana* (L.) extract (PAE) has multiple pharmacological effects, including promoting tissue repair and regeneration, anti-inflammatory (Xiao et al., 2024), anti-cancer (Lu et al., 2024), regulating gut microbiota (Cui et al., 2024), antibacterial (Liang et al., 2024), antipyretic, analgesic (Asad et al., 2024), and aiding post-stroke neural regeneration and functional recovery (Rao et al., 2024). Some of these effects align closely with current AA treatment goals, leading us to hypothesize that PAE may have therapeutic potential for AA, although no similar studies have been reported.

In this study, we first used Liquid Chromatography Tandem Mass Spectrometry (LC-MS/MS) and peptidomics to identify the main components of *P. americana* extract (PA-011). Network pharmacology was employed to predict potential targets and pathways for AA treatment. Animal experiments were conducted to evaluate its hair growth-promoting effects on AA. Skin transcriptomics, metabolomics, and 16S rRNA sequencing were performed to comprehensively analyze the relationships between skin microbiota, metabolites, differential genes, and AA. Key metabolic pathways and mechanisms were identified to reveal how PA-011 promotes hair growth in AA model mice.

## 2 Materials and methods

### 2.1 Main reagents and instruments

Sodium sulfide (Aladdin, 201417); 4% paraformaldehyde (Servicebio, 202306); Isoflurane (Shandong Ante Animal Husbandry Technology Co., Ltd., 202309); Absolute ethanol (Sinopharm Chemical Reagent Co., Ltd., 100092683); HE staining kit (Servicebio, G1003); TNF-α (Servicebio, GEM0004-48T), IL-23 (Lianke Bio, EK223-48T), and VCAM-1 (Enzyme-linked Bio, ml002068V); Wnt3a (Rabbit, A0642); Minoxidil (purchased from Beijing Nuokai Technology Co., Ltd., A04477); Dehydration machine (DIAPATH, Donatello); Embedding machine (Wuhan Junjie Electronics Co., Ltd., JB-P5); Pathological microtome (Shanghai Leica Instrument Co., Ltd., RM 2016); Tissue spreading machine (Zhejiang Jinhua Kedi Instrument Equipment Co., Ltd., KD-P); Upright optical microscope (Nikon, Japan, NIKON ECLIPSE E100); Imaging system (Nikon, Japan, NIKON DS-U3); Analytical balance (Mettler Toledo Instrument Shanghai Co., Ltd., ME203E/02); Three-button electronic digital caliper (Guilin Guanglu Digital Measurement and Control Co., Ltd., SF2000); Thermo Vanquish ultra-high-performance liquid chromatography system (Thermo Fisher Scientific, United States).

### 2.2 Extraction method of *Periplaneta americana* extract

3 kg of the dried whole bodies of adult PA (from Dali, Yunnan) were crushed. Then, four times the amount of 95% ethanol was added, and cold-soaking extraction was performed 3 times, with each extraction lasting for 3 days. The three extraction solutions were combined and filtered. The supernatant was taken and defatted with petroleum ether. The ethanol was recovered by distillation under reduced pressure at 60°C until a viscous paste was obtained. A 40%–45% ethanol aqueous solution was added, followed by stirring and standing. The mixture was kept at 4°C overnight and then filtered to remove the upper oily substances (to remove the remaining excess fat). This process was repeated three times. Subsequently, the ethanol was recovered by distillation under reduced pressure at 60°C again until a viscous paste was formed. Finally, freeze-drying was carried out to obtain the effective fraction extract of *P. americana* (L.) (PA-011).

### 2.3 Network pharmacology analysis of PA-011 for AA

#### 2.3.1 Identification of PA-011 components

Sample Treatment: Weigh 100 mg of the PA-011 sample, mix it with a methanol solution containing 4 ppm of 2-chloro-L-phenylalanine. After vortexing, grinding, and ultrasonic treatment, centrifuge at 4°C, and then filter through a 0.22-μm membrane filter.

Liquid Chromatography Conditions: Thermo Vanquish ultra-high-performance liquid chromatography system, ACQUITY UPLC^®^ HSS T3 chromatographic column. The flow rate is 0.3 mL/min, the column temperature is 40°C, and the injection volume is 2 μL. For the positive-ion mode, the mobile phase consists of 0.1% formic acid in acetonitrile and 0.1% formic acid in water; for the negative-ion mode, the mobile phase is acetonitrile and 5 mM ammonium formate in water, and the gradient elution program is shown in Supplementary Table S1. Mass Spectrometry Detection: Thermo Q Exactive mass spectrometry detector with an electrospray ionization source, collecting data in both positive and negative ion modes. The positive-ion spray voltage is 3.50 kV, the negative-ion spray voltage is-2.50 kV, the sheath gas is 40 arb, the auxiliary gas is 10 arb, the capillary temperature is 325°C. The resolution of the first-stage full-scan is 70,000, the scan range is m/z 100–1,000. HCD secondary fragmentation is carried out with a collision energy of 30 eV, the secondary resolution is 17,500. The fragmentation of the top 10 ions is collected, and useless MS/MS information is dynamically excluded.

Sample preparation: Prepare Solution A (water + 0.1% formic acid) and Solution B (80% acetonitrile + 0.1% formic acid). Dissolve the freeze-dried powder of PA-011 in 10 µL of Solution A. Centrifuge at 14,000 g for 20 min at 4°C. Take 1 µg of the supernatant as the injection sample for LC-MS detection. Liquid chromatography conditions: The elution program is shown in Supplementary Table S2. Mass spectrometry detection: Use a Q Exactive HF-X mass spectrometer with an NSI ion source. The voltage is 2.4 kV, and the tube temperature is 275°C. Employ data-dependent acquisition. Scan from m/z 100–1,500. The primary resolution is 120,000, the AGC target is 3 × 10^6^, and the maximum injection time for the C-trap is 80 m. Select the top 40 precursor ions for HCD fragmentation to measure the secondary mass spectrum. The secondary resolution is 15,000, the AGC target is 5 × 10^4^, the maximum injection time is 45 m, and the collision energy is 27%. Generate. raw data. Peptide sequence acquisition: Obtain the sequences through *de novo* analysis.

#### 2.3.2 Acquisition of targets corresponding to PA-011

Data acquisition and screening: Retrieve information about PA-011 from the PubChem database (https://pubchem.ncbi.nlm.nih.gov/). Input this information into SwissADME (http://www.swissadme.ch/index.php) to screen target compounds with a Probability >0.01. Select the top 100 sequences based on the peptide sequence-10lgP value and sequences with a nove peptid confidence >95%. Input these sequences into Emboss (https://www.ebi.ac.uk/Tools/seqstats/emboss_pepstats/) to obtain protein information. Screen sequences with a Charge value >0, resulting in 130 sequences.

Format conversion and target prediction: Use the Nover Por tool (https://www.novoprolabs.com/tools/convert-peptide-to-smiles-string) to convert the 130 polypeptide serial numbers into the SMILES format. Input the converted sequences into the Similarity ensemble approach (https://sea.bkslab.org/) to obtain predicted information on target proteins.

Information integration and duplication removal: Integrate the information on target proteins of small molecules and polypeptides. After removing duplicates, 474 pieces of target protein information are obtained.

#### 2.3.3 Acquisition of alopecia areata disease targets

Select “alopecia areata” as the search term and access the OMIM (http://www.ncbi.nlm.nih.gov/omim), GeneCards (http://www.genecards.org/), and DisGeNET (https://www.disgenet.org/search) databases to obtain Excel result files of disease-related genes. Combine the search results from the three databases. Import the disease targets and predicted polypeptide targets into the Venn 2.1.0 tool to obtain information on the intersecting genes of each polypeptide with respect to AA disease.

#### 2.3.4 Construction of the disease target interaction network (PPI)

Input the intersecting disease targets into the Search Tool for the Retrieval of Interacting Genes/Proteins (STRING) database (https://string-db.org/). Use the Cytoscape 3.9.1 software with the Centiscape 2.2 plugin to analyze the protein network. Use the default screening conditions of the plugin: Degree (>10.585), Closeness (>0.013), and Betweenness (>40.732). A total of 41 nodes and 217 edges of protein-protein interaction network information for core protein targets are obtained through screening.

#### 2.3.5 Gene ontology (GO) and kyoto encyclopedia of genes and genomes (KEGG) enrichment analysis

Import the information on the intersecting disease targets into the DAVID database (https://david.ncifcrf.gov/). Select OFFICIAL_GENE_SYMBOL in DAVID to perform GO biological process analysis and KEGG pathway enrichment analysis respectively. Use the online bioinformatics platform Bioinformatics (http://www.bioinformatics.com.cn/) to visualize the enrichment results.

#### 2.3.6 Construction of the component-pharmacodynamic target-disease pathway network

Create a network working table for the component-pharmacodynamic target-disease pathway using the obtained polypeptide target information, disease-intersecting target information, and KEGG pathway target information. Integrate the information into a tab-delimited text (TXT) file. Finally, import the data into the Cytoscape 3.9.1 software to construct a component-pharmacodynamic target-disease pathway network diagram. Simultaneously, use the Centiscape 2.2 plugin of Cytoscape 3.9.1 to calculate the node connectivity (Degree) to evaluate the relationships among drug polypeptides, pharmacodynamic targets, and disease pathways.

#### 2.3.7 Molecular docking

Perform molecular docking of the target protein with the highest correlation screened from the PPI network and the corresponding active components using the AutoDock Vina software. That is, obtain the structure files of the target protein and active components from the Protein Data Bank (PDB) database (http://wwwl.rcsb.org/). Use the PyMoL software to remove water molecules and ligands from the protein. Select the active site centered around the original ligand of the target protein for molecular docking. Finally, visualize the results using the PyMoL software.

### 2.4 Establishment of the mouse model of Alopecia areata

Fifty SPF-grade healthy female C57BL/6J mice were selected and housed in an SPF-grade environment. After 7 days of adaptive feeding, the experiment was carried out. During the experiment, the mice had free access to water and food. The experiment was divided into five groups: the blank group, the model group (using the vehicle-propylene glycol), the positive control group (2% Minoxidil), the PA-011L group (1%-PA-011), and the PA-011H group (4%-PA-011). Among them, the 1% and 4% administration doses of PA-011 were determined through preliminary pre-experiments. After all the mice were anesthetized with isoflurane, a modeling area of 3 × 4 cm was marked on the back of each mouse. Ten mice were randomly selected as the blank group (Blank), and their hair was shaved without subsequent hair-removal treatment. For the remaining mice, referring to the literature (Li et al., 2022), 6% Na_2_S was applied to their backs for hair removal. After modeling, the mice were randomly grouped and received topical administration twice a day for 21 days.

### 2.5 Observation of hair growth status and skin color scoring in mice of each group

After hair removal, the hair growth status of mice in each group was observed daily. The skin color of the mice was scored every 2 days, and the scoring criteria referred to the literature (Fu et al., 2021). Specifically, pink: 1 point; pink-white: 2 points; white: 3 points; off-white: 4 points; gray: 5 points; gray-black: 6 points. The mice in each group were photographed and recorded on the 3rd, 6th, 9th, 12th, and 18th days after hair removal.

### 2.6 Scoring of hair regeneration rate in mice of each group

After hair removal, the hair regeneration on the back was evaluated by observing the hair growth. The evaluation criteria for hair regeneration referred to the literature with slight adjustment, as follows: no growth: 0 point; 0%–20% growth: 1 point; 20%–40% growth: 2 point; 40%–60% growth: 3 point; 60%–80% growth: 4 point; 80%–90% growth: 5 point; 90%–100% growth: 6 point (Zhang et al., 2021).

### 2.7 Measurement of hair length in mice of each group

On the 15th and 21st days after hair removal, referring to the literature (Kong et al., 2022), 10 hairs were plucked from the front, middle, and back of the hair-removed area on the back of mice in each group using tweezers. The hair length was measured with a vernier caliper (in mm).

### 2.8 Weighing of hair weight in mice of each group

On the 21st day, after the mice were sacrificed, referring to the literature (Gao et al., 2023), the hair in the 3 cm × 4 cm hair-removed area was shaved off with a hair clipper and weighed using an analytical balance.

### 2.9 Detection of TNF-α, IL-23, and VCAM-1 levels in the skin tissue of mice in each group

On the 21st day, the skin samples from the hair-removed area of mice in each group were collected. The levels of TNF-α, IL-23, and VCAM-1 in the mouse skin tissue were detected according to the instructions of the Elisa kit.

### 2.10 Safety evaluation

On the 21st day, after the mice were sacrificed, the heart, liver, spleen, lung, kidney, and thymus of mice in each group were collected, weighed, recorded, and the organ indexes were calculated.

### 2.11 Observation of HE and immunofluorescence staining of mouse skin in each group

The depilated skin of mice sacrificed on the 1st and 21st days after modeling was immersed in 4% paraformaldehyde for 24 h, then embedded in paraffin, sectioned, dewaxed, and washed with water. The sections were stained with hematoxylin and eosin, photographed under a microscope, and the number of hair follicles was counted. Wax blocks were taken, sectioned, dewaxed, washed with water, subjected to citrate antigen retrieval, washed with PBS, air-dried, and then decolorized and washed with PBS three times for 5 min each time. After incubation, sections were prepared according to the instructions of the TUNEL and Ki67 kits, photographed under a fluorescence microscope, and the positive expression rate was calculated.

### 2.12 Western blot (WB) analysis

Total proteins were extracted from the skin tissue of the depilated area of mice. The skin in the depilated area of mice was washed 2–3 times with pre-cooled PBS, lysed with lysis buffer, and the total protein solution was extracted, separated by electrophoresis, and transferred to a membrane. After incubating with the primary and secondary antibodies, the membrane was washed. The relative protein expression was detected by enhanced chemiluminescence, and the images were analyzed using ImageJ (6.0) software.

### 2.13 Transcriptomic study of mouse skin in each group

Transcriptional profiles are inherently dynamic and can exhibit subtle variations even among genetically homogeneous samples due to stochastic gene expression and microenvironmental differences. Three replicate samples (n = 3 per group) provide a balance between statistical power and experimental feasibility, allowing for the detection of consistent transcriptional patterns amid such biological noise. RNA processing: total RNA was extracted from mouse skin using Trizol. The concentration, quality, and integrity were measured using a NanoDrop, and 3 μg was used as the starting amount.

Library construction: mRNA was purified using poly-T magnetic beads, fragmented with Illumina buffer and high-temperature divalent cations. First-and second-strand cDNA were synthesized, the ends were treated with enzymes and the enzymes were removed, the 3′ ends were adenylated and adapters were ligated. Fragments of 400–500 bp were selected and purified using AMPure XP, enriched by 15-cycle PCR, and then purified again using the AMPure XP system and quantified using a bioanalyzer.

Sequencing analysis: Sequencing was performed on the DNBSEQ-T7 platform. The data were subjected to GO enrichment analysis using topGO (P-value <0.05) to find the functions of differentially expressed genes, and KEGG pathway enrichment analysis using clusterProfiler (P-value <0.05).

### 2.14 Untargeted metabolomic study of mouse skin in each group

#### 2.14.1 Untargeted metabolomic analysis based on LC-MS/MS

Fifty milligrams of the skin from the depilated area of mice was weighed and ground to obtain a tissue homogenate (n = 6 per group). An ACQUITY UPLC HSS T3 chromatographic column (100 Å, 1.8 µm, 2.1 mm × 100 mm) was used, with a flow rate of 0.4 mL/min, a column temperature of 40°C, an autosampler temperature of 8°C, and an injection volume of 2 μL. For the positive and negative ion modes, mobile phase A was 0.1% formic acid in water, and mobile phase B was acetonitrile (containing 0.1% formic acid). The elution gradient is shown in Supplementary Table S3.

The Thermo Orbitrap Exploris 120 mass spectrometer, controlled by Xcalibur (4.7, Thermo), was used to acquire DDA (Data-Dependent Acquisition) mass spectrometry data in both positive and negative ion modes. An HESI (Heated Electrospray Ionization) source was employed with a spray voltage of 3.5 kV in positive mode and-3.0 kV in negative mode. After setting parameters such as the sheath gas, the primary resolution was set to 60,000, with a scan range of 100–1,000 m/z. The top 4 ions with the highest responses were selected for secondary fragmentation, with a dynamic exclusion time of 8 s. The secondary resolution was 15,000, and the HCD (Higher-Energy Collisional Dissociation) collision energy was 30%.

Both the formal samples and QC (Quality Control) samples were analyzed using the above chromatographic and mass spectrometric methods. Before injecting the formal samples, 2–4 injections of QC samples were used to equilibrate the system. During the injection process, one QC sample was injected every 5–10 formal samples for data evaluation and quality control.

#### 2.14.2 Untargeted metabolomic data processing

The R software package Ropls was used to perform dimensionality reduction analyses on the sample data, including principal component analysis (PCA), partial least squares discriminant analysis (PLS-DA), and orthogonal partial least squares discriminant analysis (OPLS-DA). Score plots were drawn to show the differences in metabolites, and permutation tests were used to prevent model overfitting. The p-value, VIP, and fold change (FC) were calculated. When the p-value was less than 0.05 and VIP was greater than 1, the metabolites were considered statistically significant. The Pheatmap (V1.0.12) in R was used to cluster the abundances of differential metabolites, and heatmaps and trend plots were drawn. The clusterProfiler (V4.6.0) was used to conduct KEGG enrichment analysis on the differential substances, and the differential abundance scores were calculated to screen the key pathways.

### 2.15 Analysis of skin microbiota diversity in mice of each group

On the 21st day, cotton swabs were used to collect samples from the depilated area of the mice’s skin. Microbial communities are characterized by inherent heterogeneity, such that even within a homogenized experimental cohort, compositional stochasticity may arise. Three biological replicates (n = 3 per group) can partially encompass such natural variation, thereby enabling robust capture of the authentic community structural features. After the samples were taken out from the refrigerator and ground, nucleic acids were extracted using the OMEGA Soil DNA Kit, and the DNA was quantified using a Nanodrop. Primers targeting the V3-V4 region of the bacterial 16S rRNA were selected for PCR amplification. The PCR products were quantified using the Quant-iT PicoGreen dsDNA Assay Kit. Libraries were constructed using Illumina technology. Qualified libraries were sequenced with 2 × 250 bp paired-end reads on an Illumina NovaSeq machine using the NovaSeq 6000 SP Reagent Kit. Part of the data analysis was completed on the GenesCloud platform of Shanghai Personal Biotechnology Co., Ltd.

### 2.16 Statistical analysis

The SPSS 19.0 statistical software was used for data analysis. The data were expressed as the mean ± standard error of the mean (SEM). The GraphPad Prism version 10 software was used for statistical analysis and plotting. One-way ANOVA was used to analyze the statistical differences among more than two groups. A P value less than 0.05 indicated a significant difference and statistical significance. Comparisons with the Model group were marked with asterisks: *p < 0.05, **p < 0.01, ***p < 0.001, ****p < 0.0001. Part of the analysis of transcriptomics, metabolomics, and microbiomics was completed on the Personal Biotechnology GenesCloud platform (https://www.genescloud.cn/analysis/diversityAnalysis).

## 3 Results

### 3.1 Identification of chemical components in PA-011

To identify the chemical components of PA-011, a combined analysis of LC-MS/MS and peptidomics was employed. Through LC-MS/MS analysis, 344 small-molecule compounds were identified. Peptidomic analysis revealed the presence of 16,515 peptide segments in PA-011. (Detailed information on the components can be found in the Supplementary Material.)

### 3.2 Acquisition of core targets

According to the LC-MS/MS analysis, PA-011 contains 344 small-molecule compounds and 16,788 polypeptide sequences, including 2,055 Database Peptides and 14,460 *De Novo* only Peptides. Based on the peptide screening criteria, 33 sequences were selected from the top 100 Database Peptides ranked by the-10lgP value, and 102 sequences were selected from the 287 *De Novo* only Peptides with a confidence greater than 95%. A total of 129 polypeptide sequences were obtained after merging and deduplication.

**FIGURE 1:**
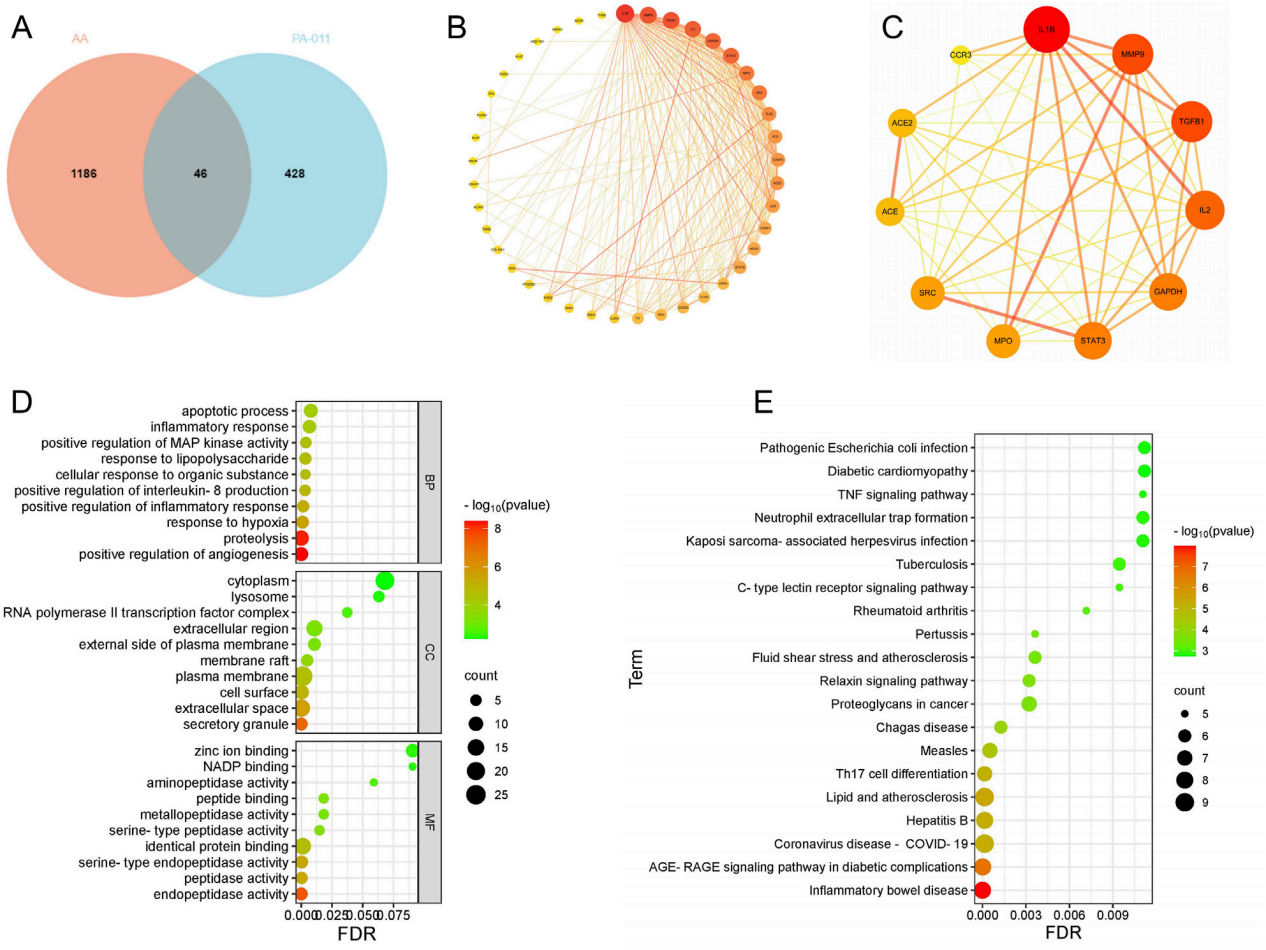
Network pharmacology analysis of PA-011 to investigate the mechanism of action of PA-011 in pemphigus vulgaris. **(A)** Venn diagram of targets between PA-011 and AA; **(B)** Intersecting targets; **(C)** Core targets; **(D)** Bubble plot of GO enrichment analysis; **(E)** Bubble plot of KEGG enrichment analysis.

By integrating the 344 small-molecule compounds and 129 polypeptide sequences of PA-011, a total of 474 drug targets were predicted. After integrating the information from disease databases, 1,232 disease targets were obtained. Analysis using a Venn diagram (Figure 1A) showed that there were 46 intersecting targets.

### 3.3 PPI analysis of core targets

The obtained intersecting targets were subjected to PPI network analysis, resulting in a PPI network diagram with 41 nodes and 217 edges (Figure 1B). According to the default screening conditions of the software plugin, a protein-protein interaction network with 11 core protein nodes and 49 edges was screened out (Figure 1C). The darker the color of the nodes, the higher the Degree value. The darker and more concentrated the color of the lines, the stronger the correlation between the proteins. Among them, core proteins such as IL-1β, IL-2, TGFB1, STAT3, and MPO, which play positive roles in the treatment of AA, show strong correlations.

### 3.4 Enrichment analysis

After performing GO enrichment analysis (Figure 1D) and KEGG enrichment analysis (Figure 1E) on the intersecting targets, the following findings were obtained:

In the biological process (BP) aspect of the GO enrichment analysis, the components of PA-011 are deeply involved in the MAP kinase cascade reaction process, positively regulate cell proliferation, precisely regulate the function of IL-8 and the process of inflammatory response, resist external stimuli, and play a positive role in key therapeutic targets for alopecia areata (AA) such as cell apoptosis regulation, inflammation response inhibition, and anti-stress response.

In the dimensions of cellular component (CC) and molecular function (MF) information, PA-011 mainly acts on inter-cellular communication and conduction, participates in cell apoptosis regulation, specifically binds to hormone signal molecules, balances the hormone levels in the body of AA patients, and achieves the effects of cell protection and anti-stimulation.

Based on the information of the main signal pathways revealed by the KEGG enrichment analysis, PA-011 can precisely regulate signal transduction pathways related to inflammatory diseases such as the TNF signaling pathway and the C-type lectin receptor signaling pathway, and induce biological processes beneficial to the treatment of AA, including anti-inflammation, anti-cell apoptosis, and optimization of cell metabolic functions.

### 3.5 Network analysis of the relationship between drug targets and pathways

**FIGURE 2:**
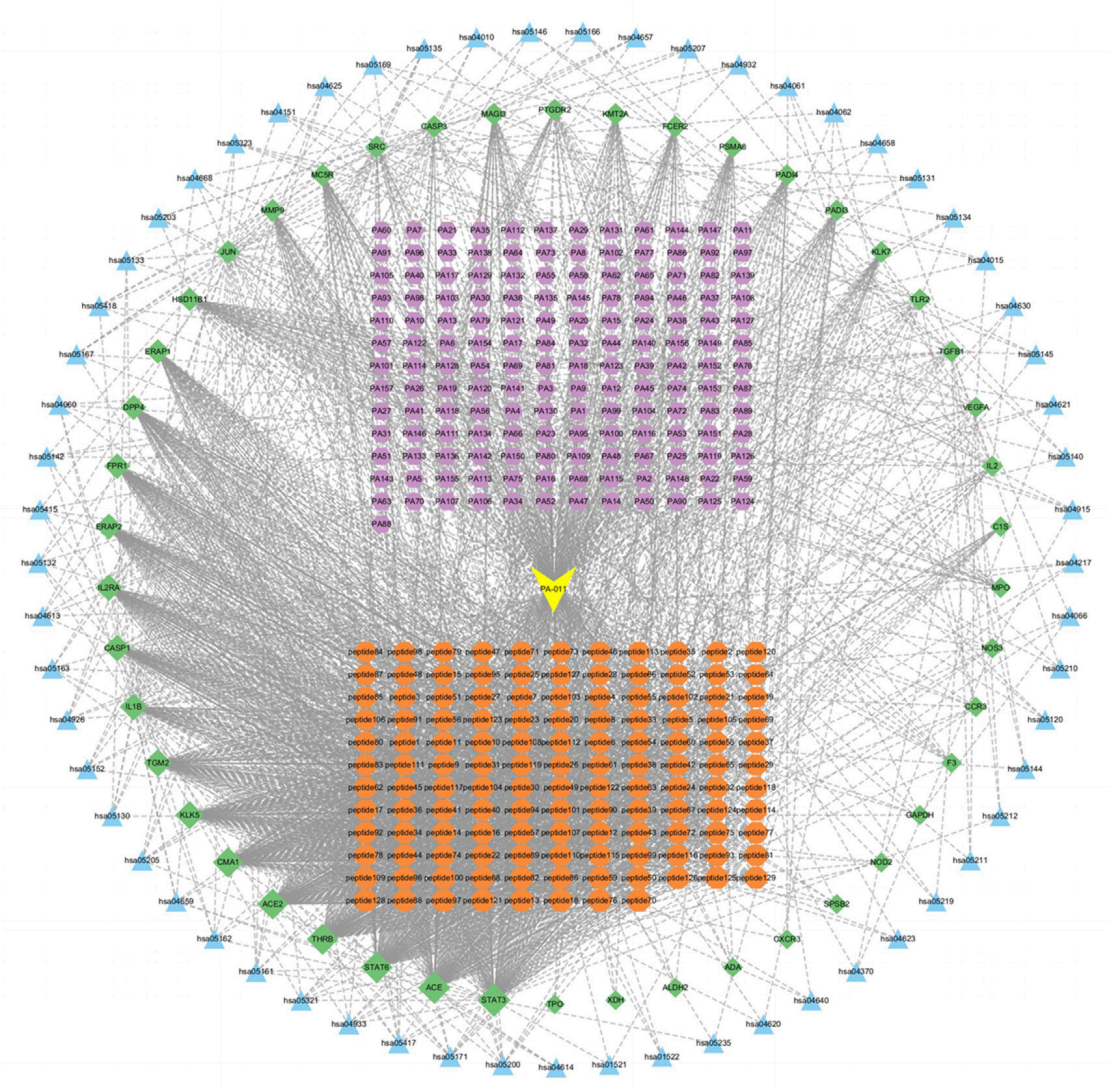
Interaction network diagram of PA-011-disease-pathway.

The collected KEGG target information, intersecting protein information, and polypeptide data were presented in a network (Figure 2). The yellow nodes represent the PA-011 samples, the orange nodes represent disease targets, the blue nodes represent small-molecule compounds, the purple nodes represent polypeptides, and the green nodes represent KEGG pathways. The close connections among these five types of nodes indicate a very high correlation between PA-011 and the therapeutic targets for AA.

### 3.6 Molecular docking

**FIGURE 3:**
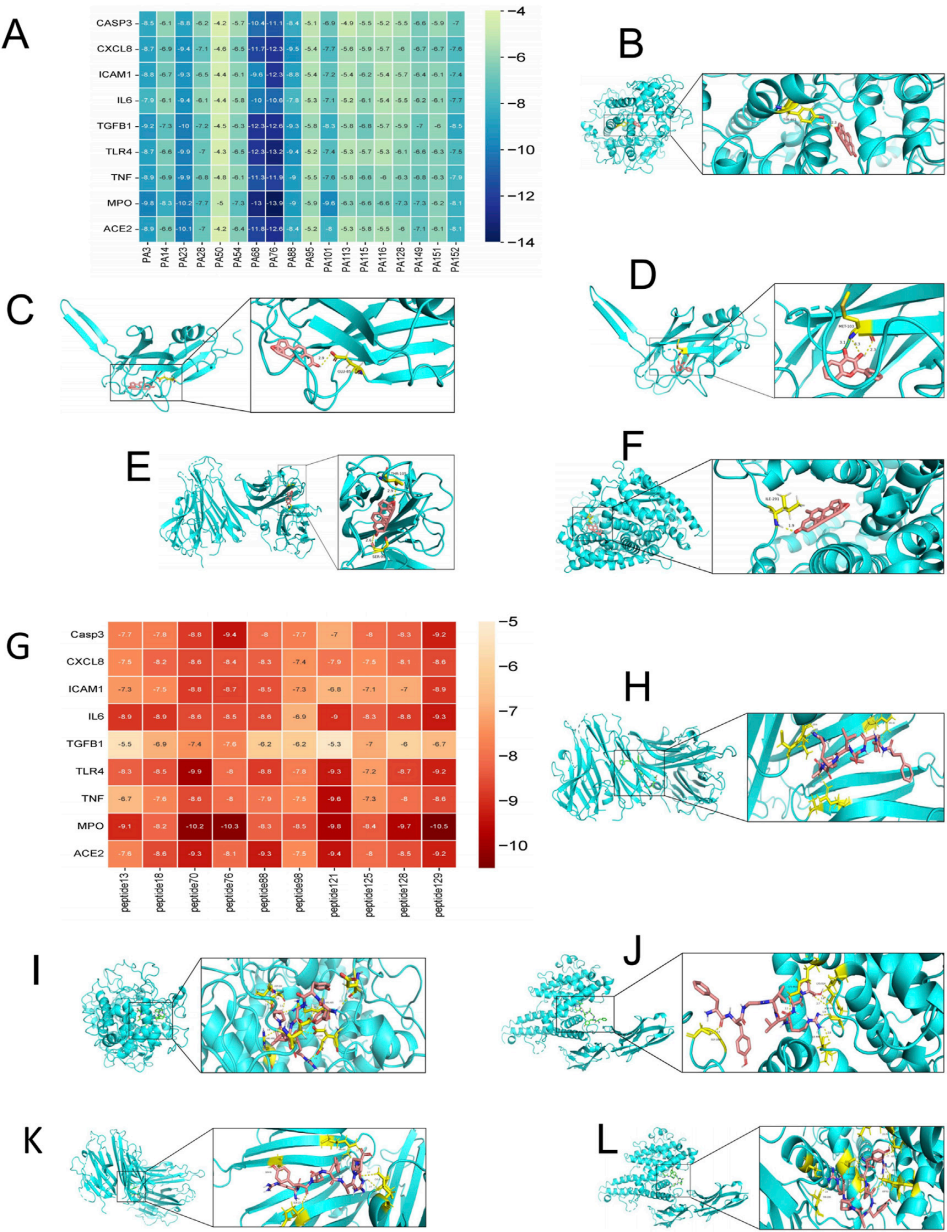
Heatmap and partial visualization of the molecular docking results of PA-011. **(A–F)** Heatmap and partial visualization of the molecular docking results of small-molecule compounds: **(B)** PA68_MPO; **(C)** PA68-TGFB1; **(D)** PA23-TGFB1; **(E)** PA68-TNF; **(F)** PA76-ACE2; **(G–L)** Heatmap and partial visualization of the molecular docking results of polypeptides: **(H)** peptide70-TNF; **(I)** peptide70-MPO; **(J)** peptide70-ICAM1; **(K)** peptide129-TNF; **(L)** peptide129-ICAM1.

Judging from the binding energy after molecular docking, the lower the binding energy between the ligand and the receptor, the more stable the binding and the stronger the binding activity. Nine target proteins with relatively high Degree values in the PPI analysis and strong associations with alopecia areata (AA) were selected for docking with 18 small molecules (Supplementary Table S4). Additionally, according to the same criteria, six target proteins were selected for molecular docking with the top 10 polypeptide sequences ranked by Degree value (Supplementary Table S5). The docking results showed that the binding energy between small-molecule compounds and their targets was less than-4.2 kcal/mol, and the binding energy between polypeptide sequences and their targets was less than-5.3 kcal/mol. As a reference, binding energy <0 kcal/mol indicates a potential interaction, with ≤−4.0 kcal/mol suggesting a strong binding affinity. The docking results were presented in a visualized heatmap (Figures 3A,G), indicating that the active components of PA-011 have strong binding activities with AA targets. Some small-molecule compounds and polypeptides with the lowest binding energies were visualized using the PyMoL software (Figures 3B–F,H–L).

### 3.7 Effects of the alopecia areata model and PA-011 on the main organs of mice in each group

**FIGURE 4:**
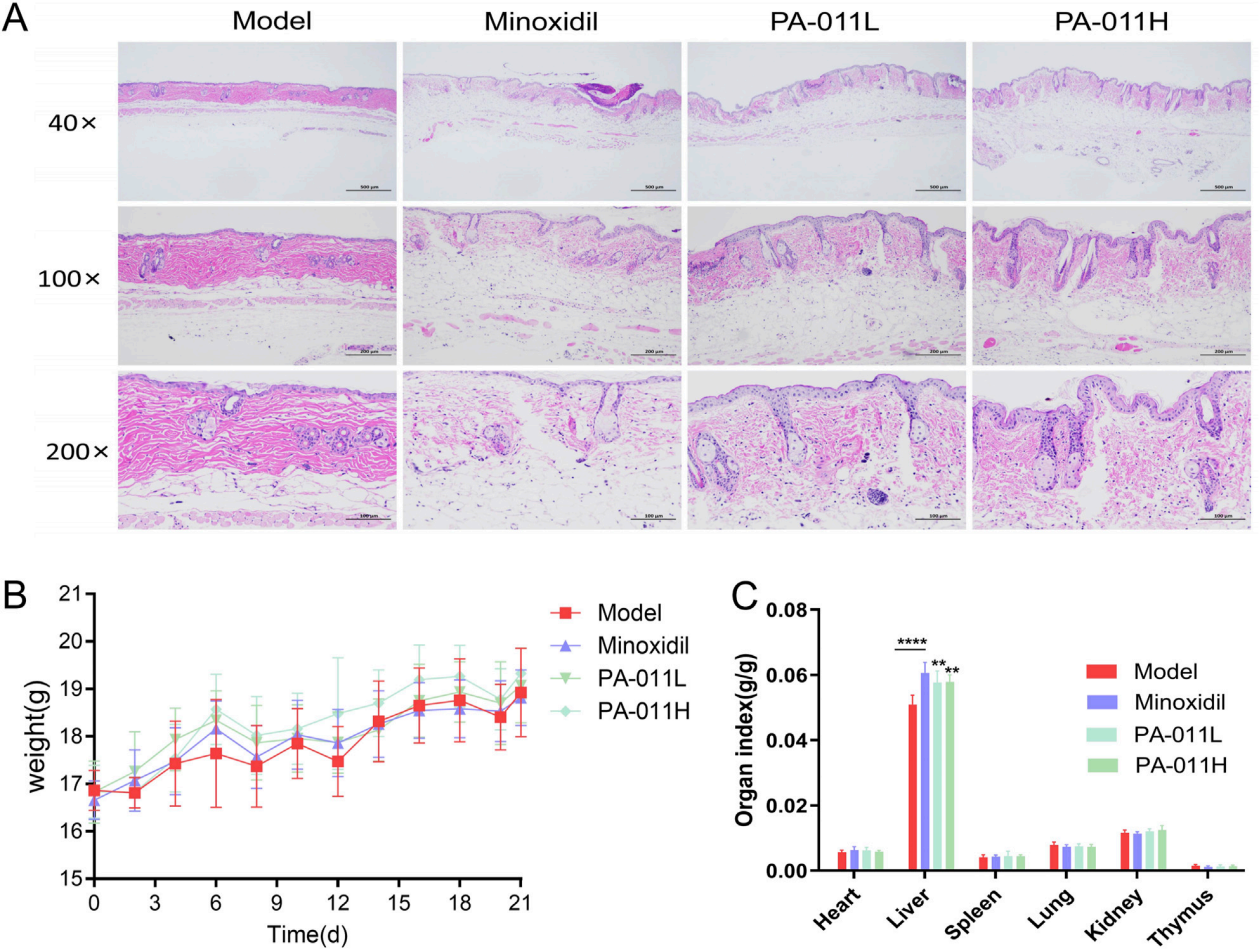
Effects of the alopecia areata model and PA-011 on the main organs of mice in each group. **(A)** HE pathological staining of the skin tissue of mice in each group after modeling (n = 1, scale bars: 500 μm, 200 μm and 100 μm); **(B)** Body weight of mice in each group (n = 9); **(C)** Organ indexes (n = 9). One-way ANOVA was used to analyze the statistical differences among more than two groups. Data are SD ± mean. **p < 0.01; ****p < 0.0001.

In AA hair follicles (HFs) usually show histological lymphocyte infiltration around and inside the hair bulb (Park et al., 2024). Pathological sections showed lesions with increased perifollicular lymphocyte infiltration (Figure 4A), indicating the successful establishment of the alopecia areata model. The body weight records of each group of mice after modeling are shown in Figure 4B. After modeling, the body weight of the mice decreased briefly, and then the body weight of each group of mice increased steadily. There were no significant differences in body weight among the four groups (p > 0.05). In addition, compared with the Model group, there were no significant differences in the organ indexes of the heart, spleen, lung, kidney, thymus, *etc.* (Figure 4C, p > 0.05). Compared with the Model group, the liver indexes were upregulated after the intervention of Minoxidil and PA-011 (p < 0.0001, p < 0.01). Alopecia areata is often accompanied by autoimmune chronic active hepatitis (AI-CAH) (Beretta-Piccoli et al., 2017). Some researchers found that after 1-year immunosuppressive treatment for alopecia areata, the condition improved significantly and the liver histology returned to normal (Sacher et al., 1990). The results indicate that PA-011 has no organ toxicity and may promote hair growth by alleviating liver damage caused by alopecia areata.

### 3.8 PA-011 can promote hair growth in AA mice

**FIGURE 5:**
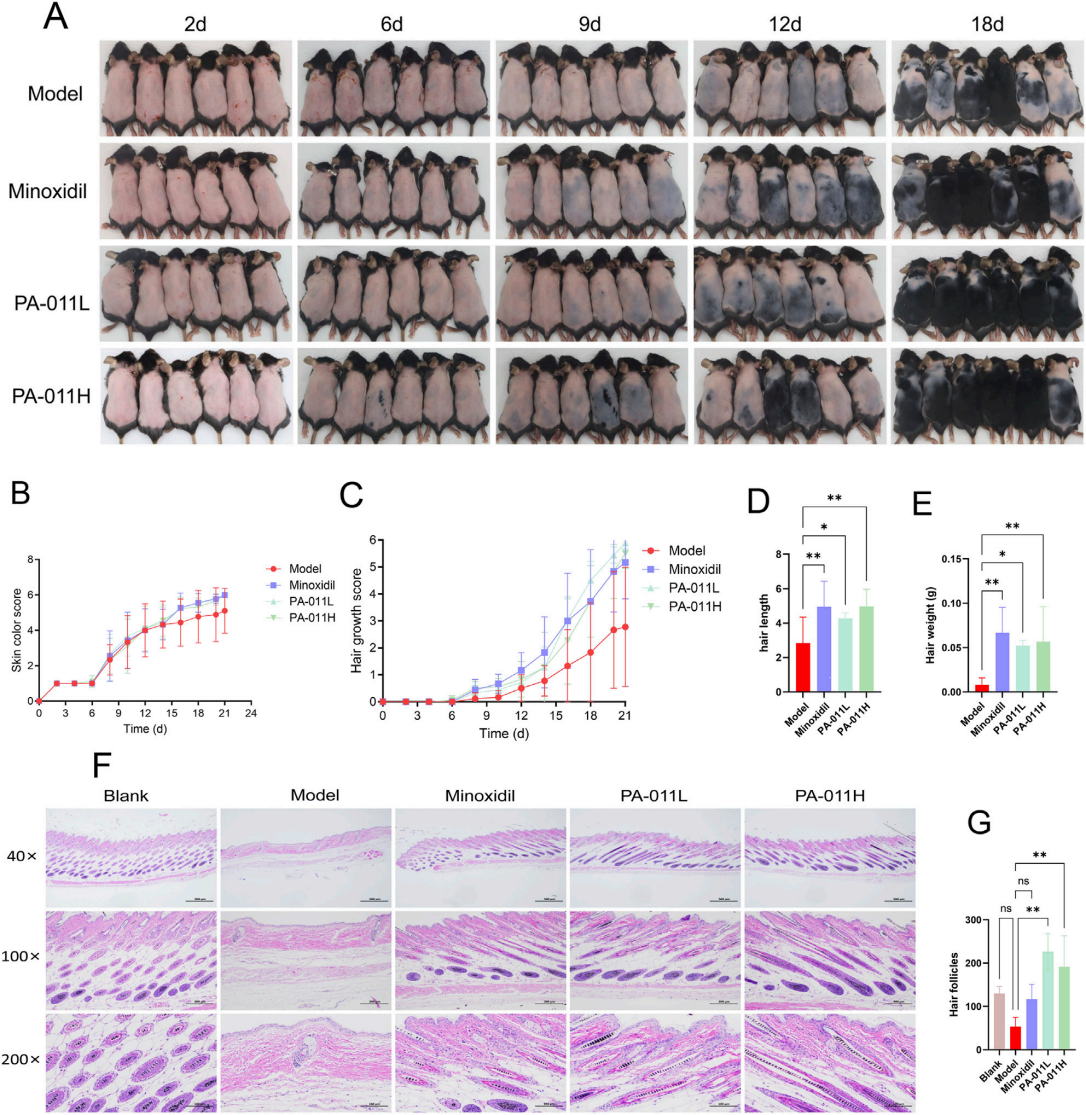
Visual observation of mouse hair. **(A)** Visual images of hair growth in each group of mice (n = 6, scale bars: 500 μm, 200 μm and 100 μm); **(B)** Skin color scores (n = 9); **(C)** Hair coverage scores (n = 9); **(D)** Hair length measurement (n = 9); **(E)** Hair weight weighing (n = 9); **(F)** HE staining of skin tissue (n = 5); **(G)** Hair follicle counting in skin tissue (n = 3). One-way ANOVA was used to analyze the statistical differences among more than two groups. Data are SD ± mean. *p < 0.05; **p < 0.01.

After constructing the AA mouse model, to explore the hair-growth promoting effect of PA-011, images of hair growth in each group of mice were collected on the 2nd, 6th, 9th, 12th, and 18th days. As shown in Figure 5A, hair regeneration in the model-group mice was blocked and the growth was slow.

After treatment with PA-011, the hair-growth rate of the mice increased (Figure 5B), and the hair coverage rate was improved (Figure 5C). This indicates that PA-011 can promote hair growth in AA mice (Figures 5D,E), and the mice enter the anagen phase earlier. The results of HE-stained pathological sections of the skin (Figure 5F) showed that both Minoxidil and PA-011 could effectively reverse the decrease in the number of hair follicles caused by AA modeling, promote hair regeneration in alopecia areata mice, and significantly increase the number of hair follicles (Figure 5G, p < 0.01), providing a pathological basis for subsequent studies.

### 3.9 PA-011 can reduce hair follicle cell apoptosis and promote hair follicle cell proliferation in the skin tissue of AA mice

**FIGURE 6:**
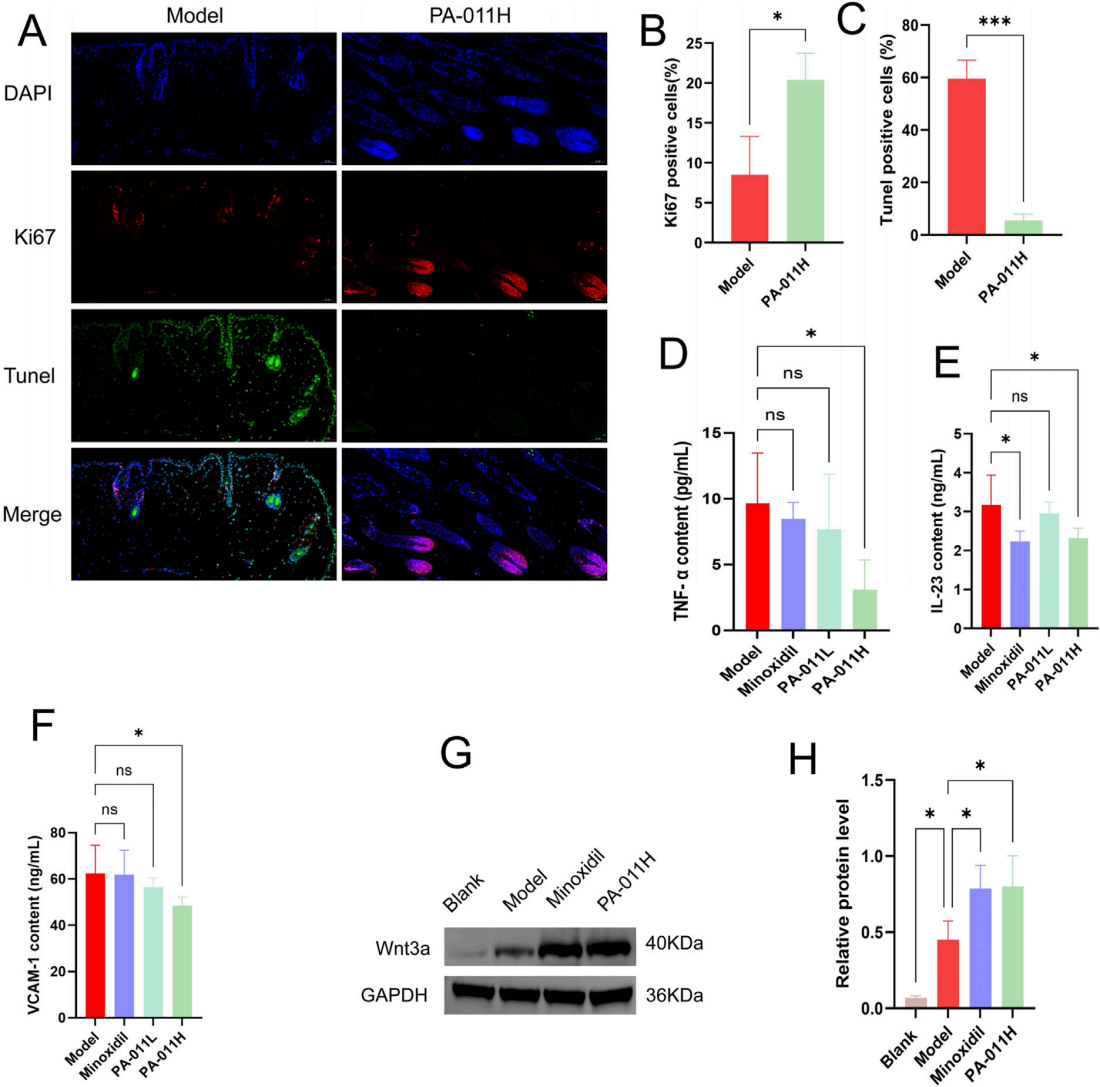
PA-011 promotes hair follicle cell proliferation, Wnt3a Protein expression and reduces Skin inflammation (n = 3–5). **(A)** Dual-channel immunofluorescence staining of Tunel and Ki67 in mouse skin tissue (scale bars: 50 μm); **(B)** Expression of Ki67-positive cells (n = 3); **(C)** Expression of Tunel-positive cells (n = 3); **(D)** TNF-α level (n = 5); **(E)** IL-23 level (n = 5); **(F)** VCAM-1 level (n = 5); **(G,H)** Verification of Wnt3a in skin tissue by Western blot (n = 3). One-way ANOVA was used to analyze the statistical differences among more than two groups. Data are SD ± mean. *p < 0.05, ***p < 0.001.

By comparing the results of skin immunofluorescence staining between the Model group and the PA-011H group (Figure 6A), it was found that treatment with PA-011 could significantly increase the expression of Ki67 (p < 0.05, Figure 6B) and significantly decrease the expression of Tunel (p < 0.001, Figure 6C). These results indicate that PA-011 can reduce the apoptosis of hair follicle cells in the skin tissue and promote their proliferation, thereby promoting hair growth in AA mice.

### 3.10 PA-011 can reduce the levels of inflammatory factors in the skin tissue of AA mice

The levels of inflammatory factors in the skin tissue of mice in each group were detected using an ELISA kit. The results are shown in Figure 8. There was low-grade inflammation in AA mice. After treatment with PA-011, the expression levels of TNF-α (Figure 6D), IL-23 (Figure 6E), and VCAM-1 (Figure 6F) in the mouse skin tissue were significantly reduced (p < 0.05), indicating that PA-011 can effectively inhibit the expression of inflammatory factors in mouse skin.

### 3.11 PA-011 promotes hair growth in AA model mice by activating the Wnt pathway

Based on the results of transcriptomics, we verified the expression level of Wnt3a protein in the Wnt pathway. The protein band diagram is shown in Figures 6G,H. After the intervention of Minoxidil and PA-011, the expression of Wnt3a was significantly increased (p < 0.05). The results indicate that PA-011 may promote hair growth in AA model mice by activating the Wnt pathway.

### 3.12 Results of transcriptomic analysis

#### 3.12.1 Analysis of gene expression levels

**FIGURE 7:**
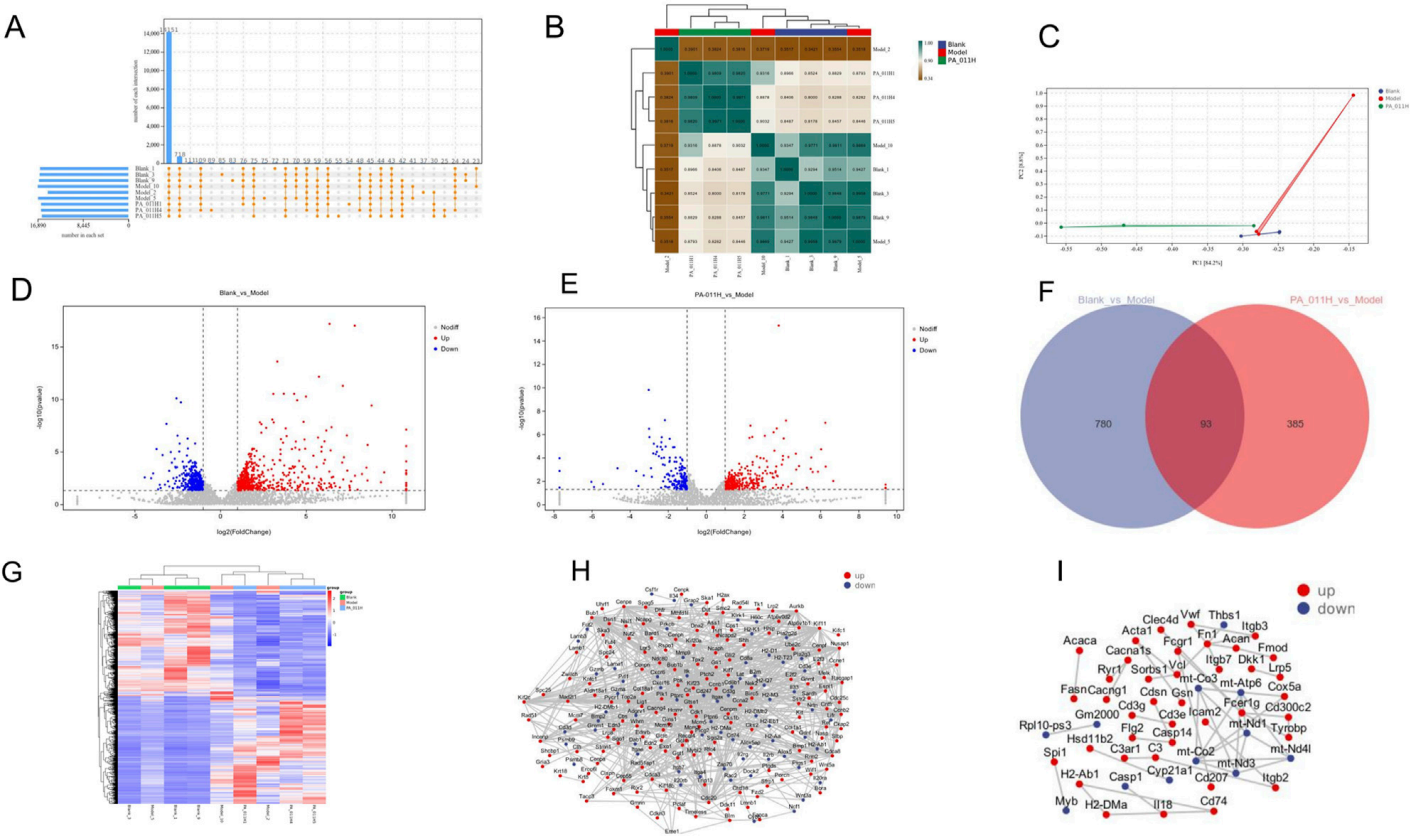
Analysis of gene expression levels and differential gene expression analysis (n = 3). **(A)** Statistics of the number of genes expressed in each group; **(B)** Correlation heatmap; **(C)** PCA analysis; **(D)** Volcano plot of differentially expressed genes in the Blank_vs._Model group; **(E)** Volcano plot of differentially expressed genes in the PA-011H_vs._Model group; **(F)** Venn diagram of differentially expressed genes in the Blank_vs._Model group and the PA-011H_vs._Model group; **(G)** Cluster heatmap of differentially expressed genes; **(H)** PPI of differentially expressed genes in the Blank_vs._Model group; **(I)** PPI of differentially expressed genes in the PA-011H_vs._Model group.

The results of transcriptomic sequencing are as follows: In the reference-based transcriptome, the gene expression levels in this experiment met the standard (FPKM >1). A total of 14,151 common genes were identified in the samples. There were 223 unique genes in the Model group that were not expressed in the PA-011 group (Figure 7A), which may be the key targets for PA-011 in the treatment of AA.

As shown in Figure 7B, the correlation coefficients were lower than 0.8 when comparing the three groups, indicating low correlations among the samples of different groups. The correlation coefficient between the samples of the PA-011H group and the Blank group was greater than 0.8, suggesting that after treatment with PA-011, the gene expression in AA mice became more similar to that in normal mice. The results of PCA analysis (Figure 7C) showed that the distances between samples of different groups were relatively large, indicating low similarity. The above results indicate that the data from the establishment of this model are reliable.

#### 3.12.2 Differential gene expression analysis

Differential analysis was performed on the expressed genes of mice in each group. There were 873 differentially expressed genes in the comparison of Blank vs. Model, with 520 upregulated genes and 353 downregulated genes (Figure 7D). In the comparison of PA-011H vs. Model, there were 478 differentially expressed genes, including 309 upregulated genes and 169 downregulated genes (Figure 7E). The two comparison groups had 93 common differentially expressed genes (Figure 7F), which may be the key gene targets of PA-011’s action on AA. The results of cluster analysis (Figure 7G) showed that there were close relationships among the three groups of samples, and treatment with PA-011 made the gene expression of AA mice closer to that of the blank group.

Protein-protein interaction analysis based on the STRING database revealed that in the Blank vs. Model comparison group, Wnt3a was downregulated and Wnt5a was upregulated (Figure 7H). In the PA-011H_vs._Model comparison group, Casp1 was downregulated and DKK1 was upregulated (Figure 7I). DKK1 is a negative regulator of the Wnt signaling pathway and inhibits hair growth in AA (Choi et al., 2024). This indicates that alopecia areata may be strongly associated with the Wnt pathway. PA-011 may upregulate the expression of the Wnt pathway, promote the proliferation of hair-follicle-related cells in the skin tissue, and thus promote hair growth.

#### 3.12.3 Functional enrichment analysis of differentially expressed genes

**FIGURE 8:**
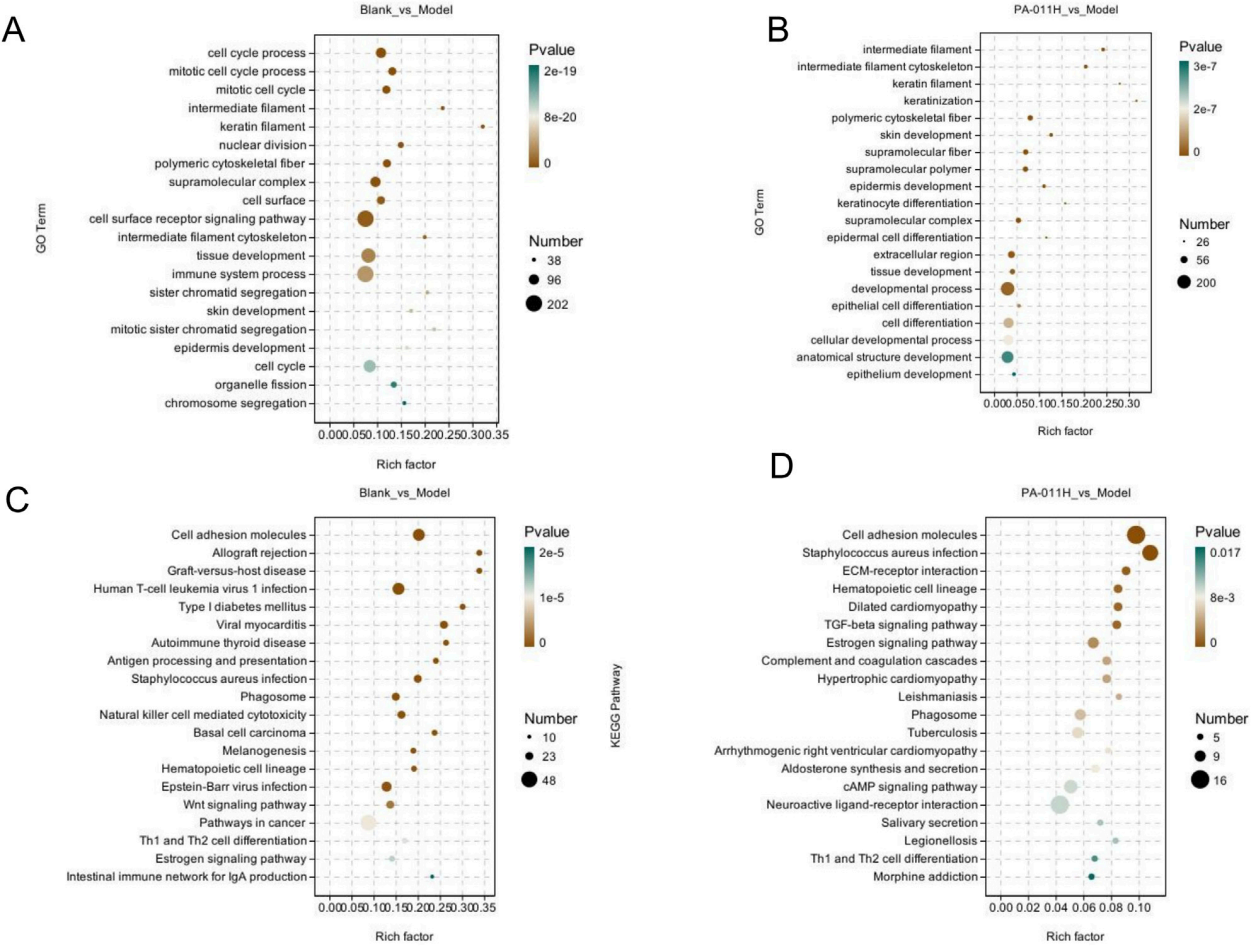
GO and KEGG enrichment analyses. **(A)** GO enrichment analysis for the Blank_vs._Model comparison group; **(B)** GO enrichment analysis for the Model_vs._PA-011H comparison group; **(C)** KEGG enrichment analysis for the Blank_vs._Model comparison group; **(D)** KEGG enrichment analysis for the Model_vs._PA-011H comparison group.

In the GO analysis, the results of Blank_vs._Model indicated that the occurrence of AA affects processes such as skin development, tissue development, and epidermal development (Figure 8A). Treatment with PA-011 has a positive regulatory effect on processes such as skin development, keratinocyte differentiation and development, and cell differentiation and development in AA mice (Figure 8B).

In the KEGG analysis, the results of Blank_vs._Model showed that the occurrence of AA affects signaling pathways such as the Wnt signaling pathway, Th1 and Th2 cell differentiation, and the Estrogen signaling pathway (Figure 8C). Treatment with PA-011 may positively regulate signaling pathways such as the TGF-beta signaling pathway, the Estrogen signaling pathway, the cAMP signaling pathway, and Th1 and Th2 cell differentiation (Figure 8D).

### 3.13 Effects of PA-011 on skin metabolism in AA mice

#### 3.13.1 Data quality control

**FIGURE 9:**
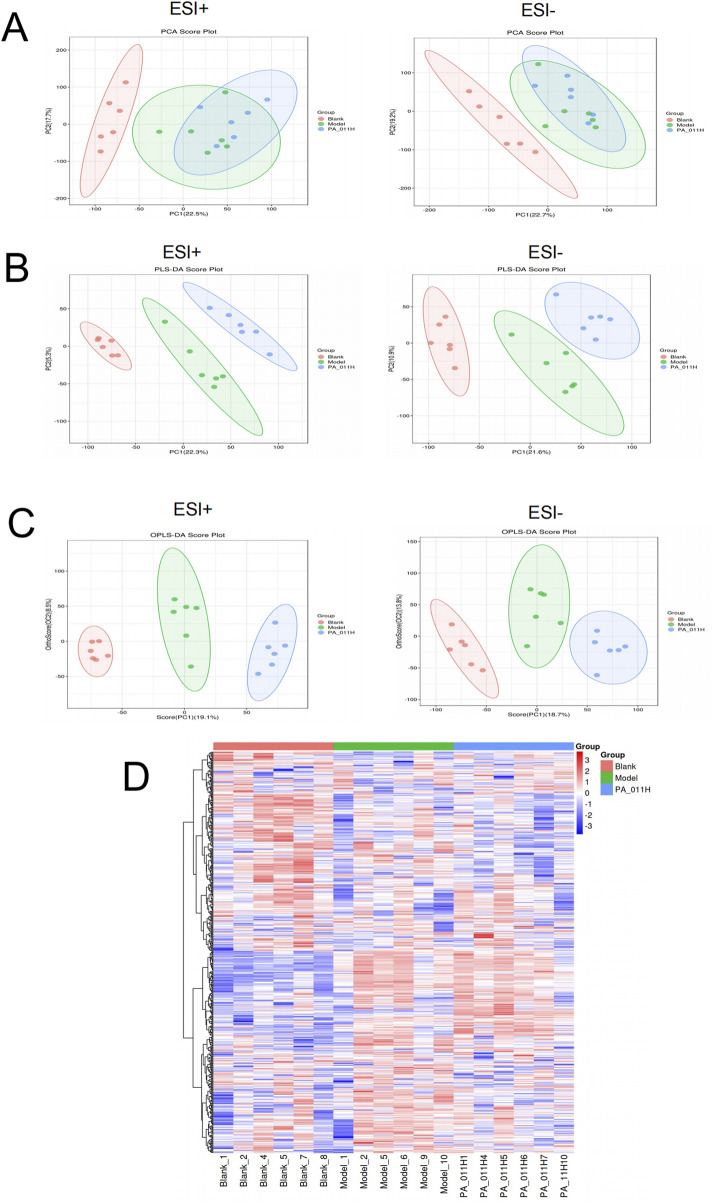
Quality control analysis of metabolic data (n = 6). **(A)** PCA analysis of positive and negative ions in each group; **(B)** PLS-DA analysis of positive and negative ions in each group; **(C)** OPLS-DA analysis of positive and negative ions in each group; **(D)** Cluster heatmap of metabolites.

Metabolomic analysis was performed on mouse skin tissue samples. PCA, PLS-DA, and OPLS-DA showed that the PA-011 group was well separated from the Blank and Model groups (Figures 9A–C). The results indicate that the model was well-constructed, and the sample classification information has good interpretive and cross-validation predictive abilities.

As can be observed from Figure 9D, there were significant differences in metabolites in the skin tissue among the three groups, and the metabolites in the skin tissue of the PA-011 group were more similar to those of the Blank group. This indicates that PA-011 can effectively improve the structural function of metabolites in the skin of AA mice, making it more similar to that of normal mice.

#### 3.13.2 Differential metabolite analysis

**FIGURE 10:**
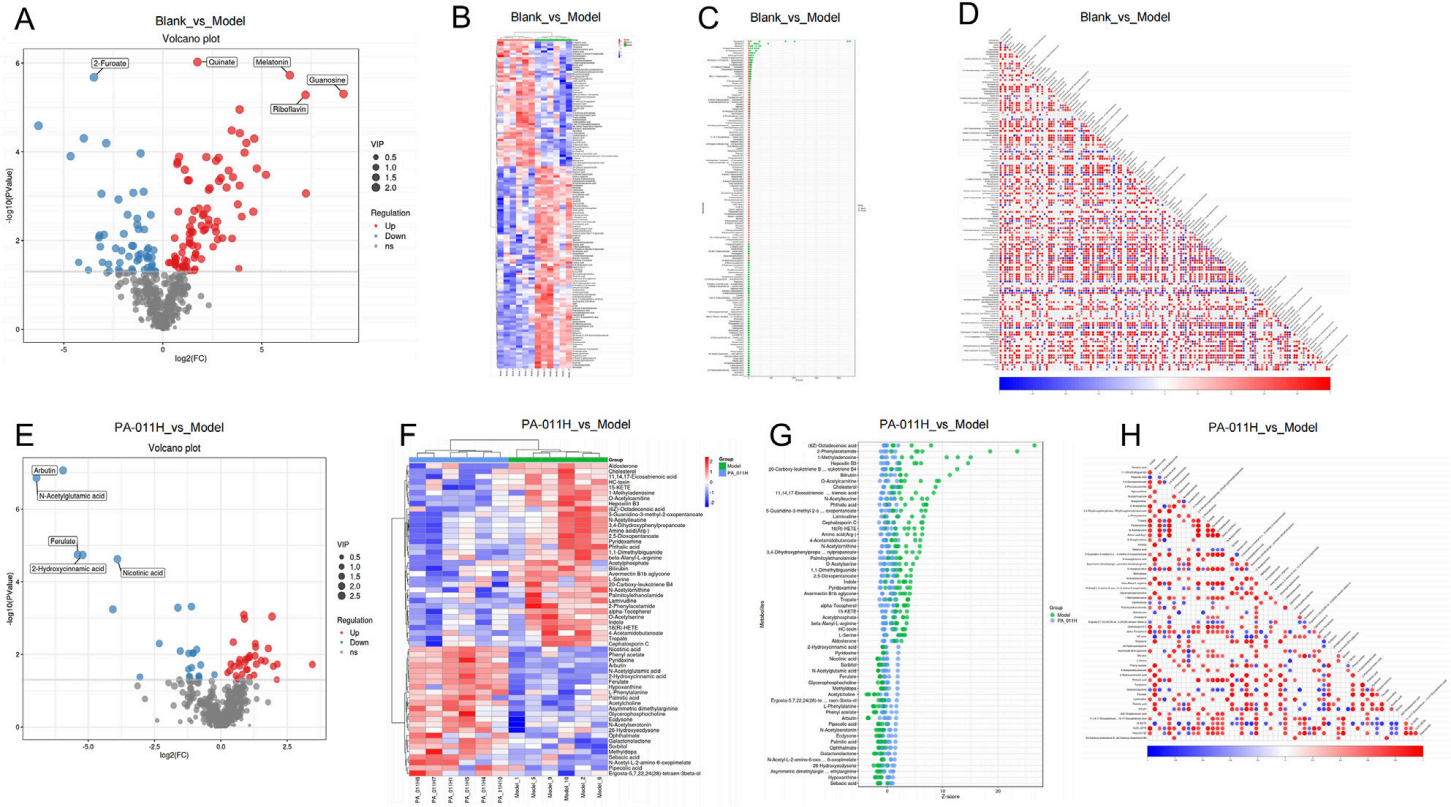
Differential metabolite analysis (n = 6). **(A)** Volcano plot of differential metabolites in the Blank_vs._Model group; **(B)** Cluster heatmap of differential metabolites in the Blank_vs._Model group; **(C)** Content score plot of differential metabolites in the Blank_vs._Model group; **(D)** Correlation analysis of differential metabolites in the Blank_vs._Model group; **(E)** Volcano plot of differential metabolites in the PA-011H_vs._Model group; **(F)** Cluster heatmap of differential metabolites in the PA-011H_vs._Model group; **(G)** Content score plot of differential metabolites in the PA-011H_vs._Model group; **(H)** Correlation analysis of differential metabolites in the PA-011H_vs._Model group.

Based on the screening criteria of variable importance in projection (VIP) >1 and p < 0.05, a total of 144 differential metabolites were identified in the Blank_vs._Model group (Figure 10A). A heatmap was generated to visualize the changes in metabolites between the two groups (Figure 10B) and the differences in content (Figure 10C). The metabolites trans-Cinnamate and L-Glutamic acid were strongly correlated and positively correlated. Trans-Cinnamate belongs to the Phenylalanine metabolism pathway, and L-Glutamic acid belongs to five pathways: Basal cell carcinoma, ABC transporters, Taste transduction, Neuroactive ligand-receptor interaction, and FoxO signaling pathway. That is, the Phenylalanine metabolism pathway is positively correlated with the FoxO signaling pathway, *etc.* Galactonolactone belongs to Galactose metabolism, and trans-Cinnamate is negatively correlated with Galactonolactone, indicating that the Phenylalanine metabolism pathway and Galactose metabolism have a negative feedback regulation (Figure 10D).

In the PA-011H_vs._Model group, a total of 58 differential metabolites were identified (Figure 10E). A heatmap was generated to visualize the changes in metabolites between the two groups (Figure 10F) and the differences in content (Figure 10G). Hepoxilin B3 is positively correlated with Bilirubin. Hepoxilin B3 belongs to the Arachidonic acid metabolism pathway, and Bilirubin belongs to Bile secretion, meaning that the Arachidonic acid metabolism pathway is positively correlated with Bile secretion. 2-Hydroxycinnamic acid is positively correlated with N-Acetylglutamic acid, and Arginine biosynthesis is positively correlated with Phenylalanine metabolism. 15-KETE is negatively correlated with Hypoxanthine, indicating that the Arachidonic acid metabolism pathway is negatively correlated with Cysteine and methionine metabolism (Figure 10H). Most metabolites were restored after PA-011 treatment, suggesting that PA-011 treatment can effectively regulate amino acid metabolism and reduce the metabolic disturbances caused by AA.

#### 3.13.3 Pathway enrichment analysis of differential metabolites

**FIGURE 11:**
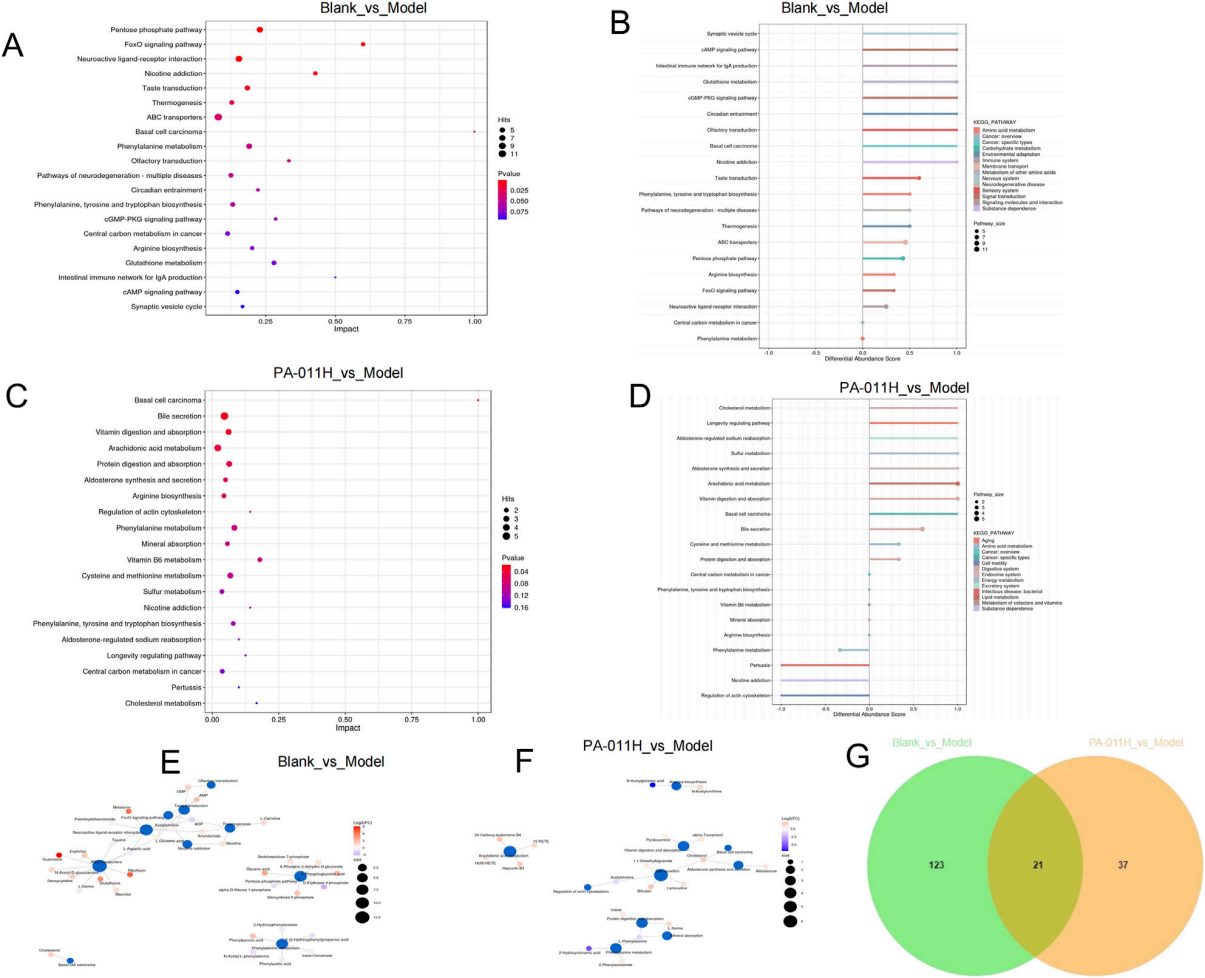
Pathway enrichment analysis of differential metabolites. **(A)** Top 20 metabolic pathways in the Blank_vs._Model group (sorted by pathway impact); **(B)** Top 20 metabolic pathways in the Blank_vs._Model group (sorted by p-value); **(C)** Top 20 metabolic pathways in the PA-011_vs._Model group (sorted by pathway impact); **(D)** Top 20 metabolic pathways in the PA-011_vs._Model group (sorted by p-value); **(E)** Metabolite-pathway network analysis of the top 10 pathways in the Blank_vs._Model group; **(F)** Metabolite-pathway network analysis of the top 10 pathways in the PA-011_vs._Model group; **(G)** Venn diagram of common metabolites between the two comparison groups.

The differential metabolites were imported into MetaboAnalyst 5.0. In the Blank_vs._Model group, 135 metabolic pathways were enriched, and in the PA-011_vs._Model group, 71 pathways were enriched. Based on a DA-score >1, the top 20 metabolic pathways in the Blank_vs._Model group included the Pentose phosphate pathway, FoxO signaling pathway, Phenylalanine metabolism, Basal cell carcinoma, Phenylalanine, tyrosine and tryptophan biosynthesis, cGMP-PKG signaling pathway, *etc.* (Figures 11A,B). The top 20 metabolic pathways in the PA-011_vs._Model group included Basal cell carcinoma, Arachidonic acid metabolism, Arginine biosynthesis, Phenylalanine metabolism, Cysteine and methionine metabolism, Phenylalanine, tyrosine and tryptophan biosynthesis, *etc.* (Figures 11C,D).

To observe the relationship between pathways and metabolites, metabolite-pathway network analysis was performed on the top 10 pathways of the two comparison groups (Figures 11E,F). Through a Venn diagram, 21 common metabolites between the two groups were screened out (Figure 11G). Based on the above results, the common metabolites in the two comparison groups and their corresponding enriched pathways suggest that PA-011 may exert an anti-AA effect by positively regulating amino acid metabolism.

### 3.14 Integrated analysis of transcriptomics and metabolomics

**FIGURE 12:**
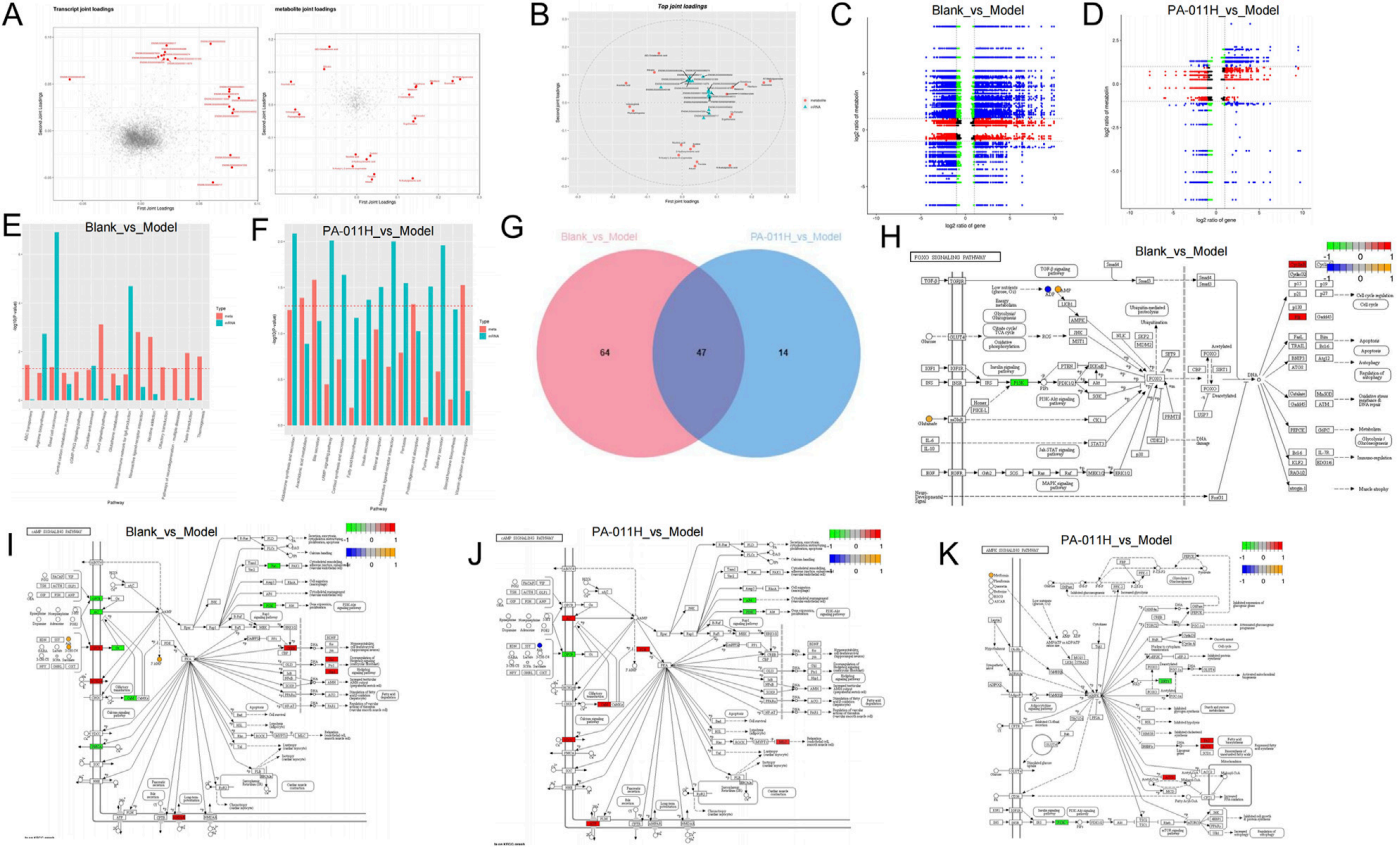
Integrated analysis of transcriptomics and metabolomics. **(A)** Loadings plot of transcriptomics and metabolomics. In A, the variables in red represent the top 20 of each omics; **(B)** Integrated loadings plot of the two omics; **(C,D)** Nine-quadrant plot of differentially expressed genes and metabolites in the Blank_vs._Model group and the PA-011H_vs._Model group; **(E,F)** Enriched pathway plots of the integrated analysis in the Blank_vs._Model group and the PA-011H_vs._Model group; **(G)** Venn diagram of enriched pathways in the two comparison groups; **(H,I)** FoxO signaling pathway and cAMP signaling pathway plots in the Blank_vs._Model group; **(J)** cAMP signaling pathway plot in the PA-011H_vs._Model group; **(K)** AMPK signaling pathway plot in the PA-011H_vs._Model group.

By plotting the loadings plot of the two omics (Figure 12A), and screening the top 20 genes and metabolites based on the sum of the squares of the loading values in the first two dimensions for integrated loading plot drawing, the genes and metabolites with the highest degree of association were found to be: Arachidic acid, Melatonin, 17α-Estradiol, Guanosine, Ferulate, H4c6, Lars2, H2bc3, Krt25 (Figure 12B). The correlation between differentially expressed genes and metabolites in each differential group was presented through a nine-quadrant plot (Figures 12C,D); the pathways co-enriched by transcriptomics and metabolomics were counted, and a bar chart was drawn to show the top 15 enriched pathways (Figures 12E,F). The results showed that after modeling, 278 pathways such as the TGF-beta signaling pathway, Wnt signaling pathway, and Th1 and Th2 cell differentiation were affected; it was found that PA-011 could effectively affect 250 pathways such as the TGF-beta signaling pathway, Estrogen signaling pathway, and cAMP signaling pathway. Among them, the Blank_vs._Model group had 111 common pathways, the PA-011H_vs._Model group had 61 common pathways, and the two comparison groups had 47 common pathways including the cAMP signaling pathway (Figure 12G). We presented the processes of the FoxO signaling pathway (Figure 12H), cAMP signaling pathway (Figure 12I) in the Blank_vs._Model group, and the cAMP signaling pathway (Figure 12J) and AMPK signaling pathway (Figure 12K) in the PA-011H_vs._Model group. The above results indicate that the occurrence of alopecia areata may be related to pathways such as the cAMP signaling pathway, AMPK signaling pathway, and FoxO signaling pathway, and the administration of PA-011 can effectively act on the above-mentioned pathways to reduce skin inflammation, coordinate cell growth, autophagy, and metabolism, and exert a hair-promoting effect.

### 3.15 Effects of PA-011 on the skin microbiota of AA mice

#### 3.15.1 PA-011 can effectively adjust the skin microbial diversity of AA mice

**FIGURE 13:**
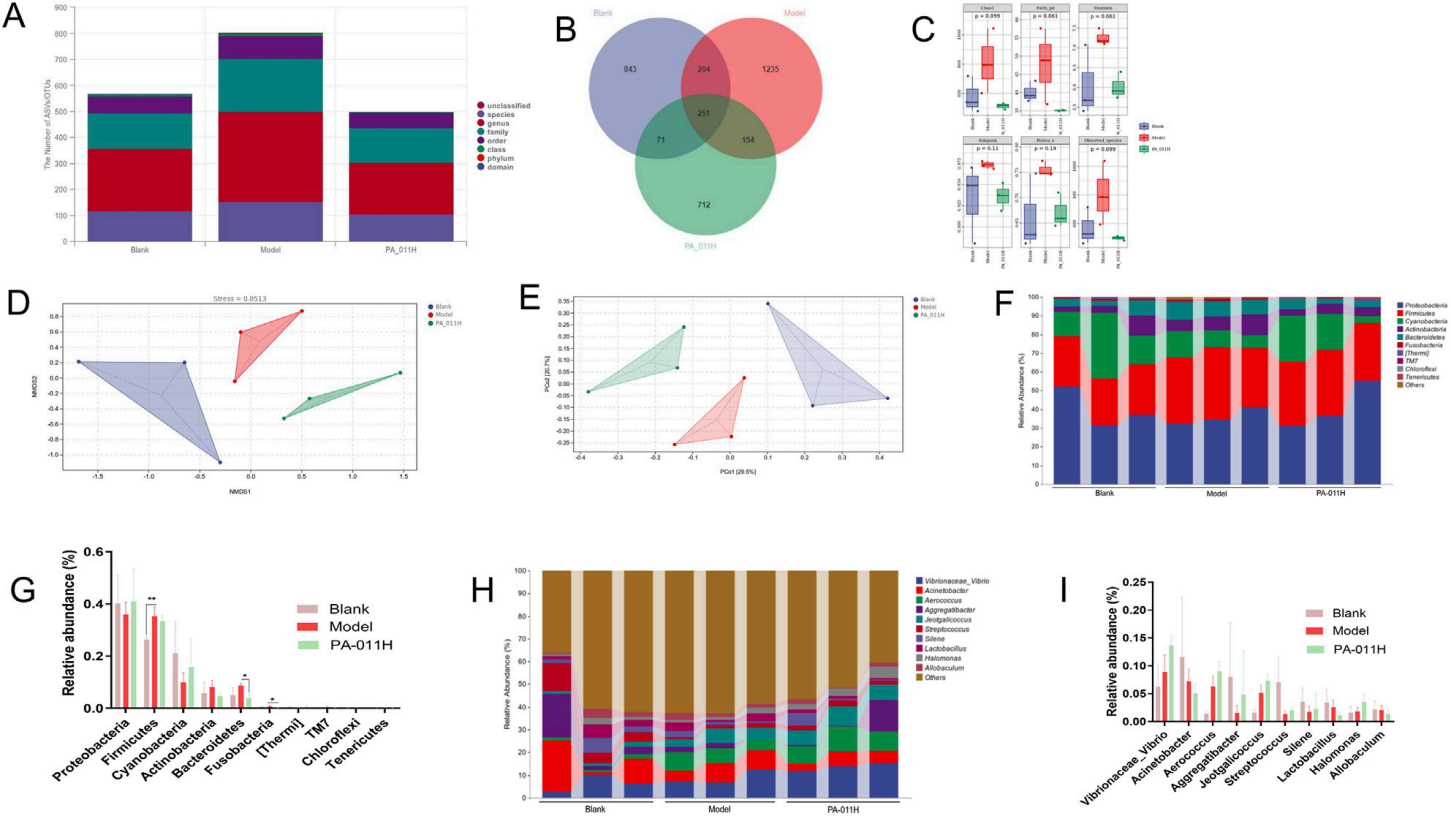
PA-011 can regulate the skin microbial diversity of AA model mice (n = 3). **(A)** Microbial OTUs at each taxonomic level; **(B)** Venn diagram of microbial OTUs in each group; **(C)** Alpha diversity analysis; **(D)** NMDS analysis; **(E)** PCoA analysis; **(F,G)** Taxonomic composition analysis at the phylum level; **(H,I)** Taxonomic composition analysis at the genus level.

For 16S rRNA sequencing, in the composition of skin microbiota, there were 33 OTUs at the phylum level, 74 OTUs at the class level, 134 OTUs at the order level, 266 OTUs at the family level, 633 OTUs at the genus level, and 46 OTUs at the species level (Figure 13A). The Venn diagrams of each group also showed that after the intervention of PA-011, the structure of the skin microbial flora was closer to that of the blank group (Figure 13B). The Chao1, Observed species, Shannon, Simpson, Faith’s PD, and Pielou’s evenness indices indicated that the occurrence of AA significantly changed the richness and diversity of the skin microbiota in mice, while the treatment with PA-011 could make the ecological structure of the flora closer to that of the blank group (Figure 13C). In the non-metric multidimensional scaling analysis (NMDS), Stress = 0.0513 (Figure 13D), and in the principal coordinates analysis (PCoA), the distances between each group were relatively far (Figure 13E), indicating that there were significant differences in microbial species among each group and the data were reliable.

Through the analysis of the abundance percentages of the top 10 microorganisms at the phylum level (Figures 13F,G) and genus level (Figures 13H,I), it was found that PA-011 could effectively increase the abundance levels of probiotics such as Proteobacteria, Cyanobacteria, and Aggregatibacter. At the same time, it reversed the increase in the abundance levels of pathogenic bacteria such as Bacteroidetes, Fusobacteria, and Actinobacteria caused by AA. This indicates that PA-011 can effectively increase the abundance of probiotics beneficial to the anti-AA effect and improve the damage to the ecological structure of the skin microbiota caused by AA.

#### 3.15.2 Functional prediction of differential skin microorganisms

**FIGURE 14:**
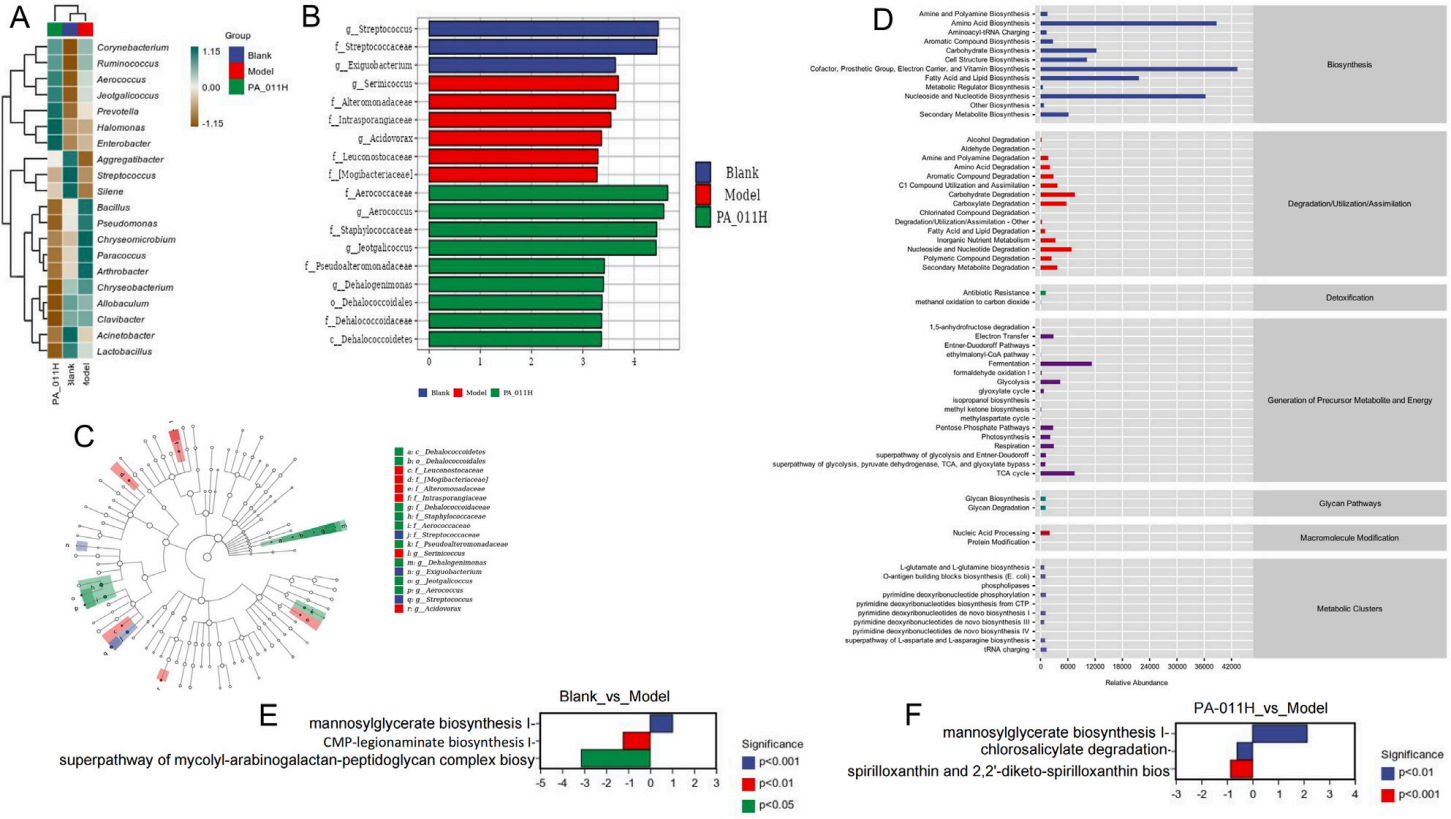
Functional difference prediction of differential skin microorganisms. **(A)** Heat map analysis of the abundance of mouse skin microorganisms; **(B)** LDA bar chart; **(C)** LDA cladogram; **(D)** KEGG functional prediction analysis; **(E)** Differential pathways between Blank and Model; **(F)** Differential pathways between PA-011H and Model.

Through the heat map analysis of the microorganisms in the three groups, it can be seen that compared with normal mice, the microbial composition of AA mice is quite different, and the composition of the microbial community changes after PA-011 treatment (Figure 14A). From the LEfSe analysis, the dominant microorganisms with LDA scores in the Blank group are g_*Streptococcus*; those in the Model group are g_Serinicoccus; and those in the PA-011 group are f_Aerococcaceae and g_Aerococcus (Figures 14B,C).

KEGG functional prediction analysis was carried out on the functions of differential microorganisms (Figure 14D), and 60 enriched pathways were obtained. The main functions include amino acid biosynthesis, biosynthesis of electron carriers and vitamins, biosynthesis and degradation of nucleosides and nucleotides, pentose phosphate pathway, biosynthesis of L-glutamate and L-glutamine, and other biological function pathways. In the comparison of functions between Blank_vs._Model and PA-011_vs._Model, it was found that the occurrence of AA significantly affects biological processes such as mannosylglycerate biosynthesis I, CMP-legionaminate biosynthesis I, and superpathway of mycolyl-arabogalactan-peptidoglycan complex biosynthesis (Figure 14E). However, after treatment with PA-011, the biological function processes can be changed, significantly affecting biological processes such as mannosylglycerate biosynthesis I and chlorosalicylate degradation (Figure 14F). The results suggest that PA-011 may play an anti-AA functional role by regulating high-quality skin microbial strains, thus influencing a variety of biological function processes.

### 3.16 Integrated analysis of transcriptomics, metabolomics, and skin microbiota

**FIGURE 15:**
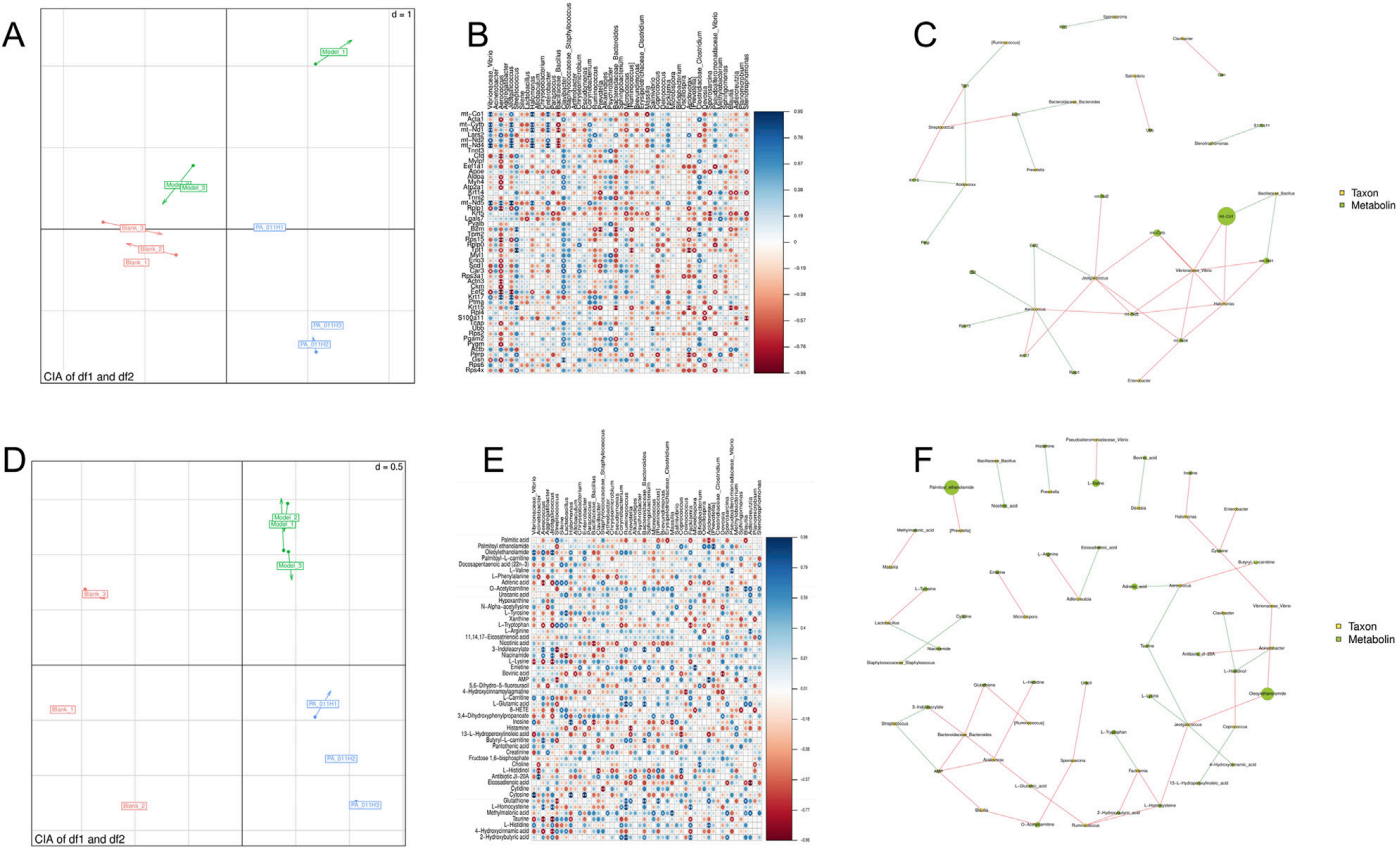
Integrated analysis of transcriptomics, metabolomics, and skin microbiota. **(A)** Canonical Inertia Analysis (CIA) of skin microbiota and transcriptomics; **(B,C)** Spearman correlation analysis between skin microbiota at the genus level and transcriptomic data; **(D)** Canonical Inertia Analysis (CIA) of skin microbiota and metabolomics; **(E,F)** Spearman correlation analysis between skin microbiota at the genus level and metabolomic data.

A Canonical Inertia Analysis (CIA) was performed on the composition profile of skin microbial diversity at the genus level and transcriptomic data. The RV value was 0.6 (Figure 15A), indicating a high degree of correlation between skin microbiota and skin transcriptomics. Additionally, using Mothur software, the Spearman rank correlation coefficient between transcriptomic data and microbial abundance was calculated. Analyzing the correlation information with |rho| >0.8 and P-value <0.01, it was found that Vibrionaceae_*Vibrio* was positively correlated with mt-Co1, mt-Cytb, mt-Nd1, mt-Nd4, and mt-Nd5; Jeotgalicoccus was positively correlated with mt-Cytb, mt-Nd2, mt-Nd4, mt-Nd5, and Krt17; Aerococcus was positively correlated with mt-Nd5 and Krt17; *Streptococcus* was positively correlated with B2m, Tpt1, and Krt15; Halomonas was positively correlated with mt-Co1, mt-Cytb, mt-Nd1, mt-Nd2, mt-Nd4, and mt-Nd5; *Enterobacter* was positively correlated with mt-Co1, mt-Cytb, mt-Nd1, and mt-Nd4; Clavibacter was positively correlated with Gsn; Aerococcus was negatively correlated with Rplp1, Rps15, Actn3, and Eef2; Jeotgalicoccus was negatively correlated with Eef2; Bacillaceae_*Bacillus* was negatively correlated with mt-Co1, mt-Nd1, mt-Nd2, and mt-Nd4 (Figures 15B,C).

CIA was also carried out on the composition profile of skin microbial diversity at the genus level and metabolomic data (Figure 15D). The RV value was 0.857, P < 0.01, indicating a high degree of correlation between skin microbiota and metabolomics. Vibrionaceae_*Vibrio* was positively correlated with Oleoylethanolamide and Cytosine, and negatively correlated with L-Lysine; Jeotgalicoccus was positively correlated with Oleoylethanolamide, Niacinamide, and L-Homocysteine, and negatively correlated with Taurine and 4-Hydroxycinnamic acid; Aerococcus was positively correlated with 3-Indoleacrylate, Butyryl-L-carnitine, and L-Homocysteine, and negatively correlated with Adrenic acid and Taurine; *Streptococcus* was negatively correlated with 3-Indoleacrylate, AMP, and Glutathione; *Lactobacillus* was positively correlated with L-Tyrosine and negatively correlated with Niacinamide; Halomonas was positively correlated with Inosine and Cytosine; Bacillaceae_*Bacillus* was negatively correlated with Nicotinic acid and Inosine; Ruminococcus was positively correlated with L-Glutamic acid, AMP, L-Glutamic acid, Butyryl-L-carnitine, and Glutathione; Bacteroidaceae_*Bacteroides* was positively correlated with 3-Indoleacrylate, AMP, L-Glutamic acid, Butyryl-L-carnitine, and Glutathione; Coprococcus was positively correlated with L-Histidinol and negatively correlated with 13-L-Hydroperoxylinoleic acid; Facklamia was positively correlated with L-Homocysteine and 2-Hydroxybutyric acid, and negatively correlated with L-Tryptophan; Acidovorax was positively correlated with AMP and L-Histidine; Adlercreutzia was positively correlated with L-Arginine and negatively correlated with Eicosadienoic acid; Blautia was positively correlated with O-Acetylcarnitine and AMP (Figures 15E,F).

Analysis of the pathways to which the related genes and metabolites belong shows that PA-011 exerts its hair-promoting effect mainly by regulating mitochondrial-related functions, the Estrogen signaling pathway, and amino acid metabolism.

## 4 Discussion

In alopecia areata (AA), inflammation is a primary trigger that has garnered significant attention. Epidemiological data indicate that the lifetime risk of developing AA in the general population is approximately 2.1% (Lee et al., 2022). The pathogenesis involves inflammation triggering complex signaling cascades, which strongly drive immune cell infiltration into skin tissue, leading to the collapse of the hair follicle (HF) immune privilege and ultimately causing severe damage to HFs in AA patients (Gund and Christiano, 2023). Histologically, AA lesions are characterized by dense perifollicular and intrafollicular inflammatory cell infiltration, forcing HFs into premature regression and even inducing apoptosis in severe cases (He et al., 2024). With rising societal stress and profound lifestyle changes, the incidence of AA has shown an increasing trend in recent years (Ly et al., 2023). Therefore, identifying and developing potential therapeutic agents for AA holds significant practical importance.

In this study, we employed network pharmacology to predict the targets and pathways of *Periplaneta americana* extract PA-011 in AA treatment. We identified 18 small-molecule compounds (e.g., (R)-Methysticin, 2-Phenylacetamide, and 3-Hydroxyflavone) and 10 peptides (e.g., FQQRPQPQPQPQPQ, FYGVVRAP, and TPFYLR), whose core targets primarily focus on key factors such as IL-1β, IL-2, TGFB1, STAT3, and MPO. GO and KEGG enrichment analyses revealed that these components exert anti-AA effects through anti-inflammatory and apoptosis-inhibiting pathways.

In animal experiments, PA-011 intervention significantly improved hair regrowth in AA mice, with higher hair coverage compared to untreated controls. Studies suggest that immune system dysfunction leads to loss of immune tolerance to HF-specific antigens, triggering intense immune and inflammatory responses that disrupt HF growth, resulting in AA (Dahabreh et al., 2023). Consistently, H&E staining of skin histopathology showed a marked reduction in HF numbers in AA mice compared to healthy controls, which was significantly reversed by PA-011 treatment. Immunofluorescence assays further demonstrated that PA-011 reduced TUNEL expression (indicating apoptosis) and enhanced Ki67 expression (a proliferation marker) in HFs, confirming its role in mitigating AA-induced HF damage.

Additionally, studies report elevated serum levels of Th1, TNF-α, and IL-23 in AA patients compared to healthy individuals, alongside reduced expression of Wnt/β-catenin signaling factors (Park et al., 2024; Waśkiel-Burnat et al., 2021). The biomarker vascular cell adhesion molecule-1 (VCAM-1) in AA recruits immune cells, promotes inflammation, releases pro-inflammatory factors, and impairs microvascular function (Luo et al., 2024). Our findings revealed that PA-011 significantly reduced IL-23, TNF-α, and VCAM-1 levels in AA mouse skin, aligning with network pharmacology predictions and confirming its anti-inflammatory efficacy.

Transcriptomic analysis showed that PA-011-induced differentially expressed genes were enriched in pathways such as Wnt signaling, Th1/Th2 differentiation, TGF-beta signaling, estrogen signaling, and cAMP signaling. Notably, Wnt3a and Wnt10b, key members of the Wnt family, play critical roles in HF cycle regulation and regeneration by promoting anagen gene expression (Lee et al., 2021). Recent studies highlight that Wnt3a overexpression counteracts Wnt5a-mediated inhibition of cell proliferation, favoring HF regeneration (Chang et al., 2024). WB results confirmed significant upregulation of Wnt3a protein after PA-011 treatment, consistent with transcriptomic data.

Metabolomic analysis indicated that PA-011 exerts anti-AA effects by modulating the pentose phosphate pathway, FoxO signaling, phenylalanine metabolism, basal cell carcinoma-related pathways, and cGMP-PKG signaling. Inhibition of the pentose phosphate pathway promotes hair growth (Badolati et al., 2018), while FoxO signaling regulates proliferation, cell cycle arrest, and apoptosis (Zhang and Zhang, 2019). Basal cell carcinoma pathways involve Wnt activation, with upregulated Wnt-related genes linked to HF growth (Dessinioti et al., 2017). These findings suggest that PA-011 suppresses pentose phosphate synthesis, regulates amino acid metabolism, and enhances Wnt signaling to combat AA.

Under normal physiological conditions, HFs possess immune privilege, maintained by their unique anatomy and cellular composition, which ensures stable growth and cyclical renewal (Eisenstein, 2020). The skin microbiome interacts intricately with the host, producing antimicrobial compounds (e.g., bacteriocins, proteases) and metabolites (e.g., short-chain fatty acids, vitamins) that suppress pathogens and support HF health (Glatthardt et al., 2024). Dysbiosis disrupts this balance, triggering immune dysregulation and AA (Stacy and Belkaid, 2019; Belkaid and Tamoutounour, 2016).

Severe AA patients exhibit altered scalp microbial communities, with microbial metabolites profoundly influencing disease progression. Among them, short-chain fatty acids (SCFAs), metabolites of some microorganisms, have attracted much attention in recent years in the field of skin microecology, which can inhibit or destroy harmful bacteria and modulate the host immune system (Park et al., 2021). In contrast, some dermal symbiotic bacteria are able to synthesize vitamin A, vitamin B, vitamin K, and other vitamins, which are essential nutrients for maintaining normal metabolism and physiological functions of hair follicle cells (Roche and Harris-Tryon, 2021; Joshi et al., 2023). For instance, Propionibacterium species produce antimicrobial substances (e.g., propionic acid) to suppress inflammation (Kang et al., 2015), while overgrowth of Malassezia releases pro-inflammatory lipids and cytokines (e.g., IL-1β, TNF-α), exacerbating AA (Grice and Dawson, 2017). Our microbiome analysis revealed elevated pathogenic genera (Malassezia, Alteromonadaceae, Acidovorax, Leuconostocaceae) in AA mice, which PA-011 treatment effectively reduced while enriching beneficial taxa (Aerococcaceae, Staphylococcaceae, Jeotgalicoccus, Pseudoalteromonadaceae). Aerococcaceae and Staphvlococcaceae serve as core species in the skin microbiota, and they are importantly linked to skin inflammation and wound healing (Rossi, 2023). Functional predictions suggest PA-011 restores microbial diversity, modulates mitochondrial function, estrogen signaling, and amino acid/nucleotide metabolism to suppress autoimmunity and inflammation.

Integrated multi-omics analyses (transcriptomics, metabolomics, microbiome) demonstrate that PA-011 may promotes hair regeneration by activating Wnt signaling, suppressing inflammatory pathways, and restoring microbial balance. However, further studies—including targeted inhibition of key signaling molecules, pathway-specific agonist/antagonist-mediated rescue assays, and *in vivo* validation using genetically modified models—are required to delineate the precise mechanisms through which PA-011 counteracts AA, encompassing its anti-inflammatory effects, regulation of microbial activity, and modulation of metabolite production.

## 5 Conclusion

PA-011 may involve its anti-AA effect through the following mechanisms. It activates the expression of Wnt3a, regulates the TGF-β signaling pathway, inhibits inflammatory genes, reduces the expression of IL-23, TNF-α, and VCAM-1 in the skin, thereby alleviating skin inflammation. Meanwhile, it modulates the ecological structure of the skin microbiota, which in turn changes the metabolites of the microbiota, improves the ecological micro-environment of hair follicles, and promotes the proliferation of hair-follicle cells. Therefore, PA-011 can be further investigated as a potential therapeutic drug for AA.

## Data Availability

The data presented in the study are deposited in the Mendeley Data repository, available at: https://data.mendeley.com/datasets/7mncdjvwpb/1 (DOI: 10.17632/7mncdjvwpp.1).
