# Supplementary material for: Periplaneta americana extract (L.) promotes hair regrowth in Alopecia areata mice by reducing inflammation and modulating skin microbiota
Source: Front Pharmacol. 2025 Aug 6;16:1590648. doi: 10.3389/fphar.2025.1590648 (PMC12365694; doi:10.3389/fphar.2025.1590648)
Supplement: Supplementary file 1 [file Supplementaryfile1.docx]

**Table S1.** PA-011 small molecule gradient elution program

| **Time** | | **Velocity of flow** |
| --- | --- | --- |
| POS mode | 0-1 min | 8% B2 |
|  | 1-8 min | 8%-98% B2 |
|  | 8-10 min | 98% B2 |
|  | 10-10.1 min | 98%-8%B2 |
|  | 10.1-12 min | 8% B2 |
| NEG mode | 0-1 min | 8% B3 |
|  | 1-8 min | 8%-98% B3 |
|  | 8-10 min | 98% B3 |
|  | 10-10.1 min | 98%-8% B3 |
|  | 10.1~12 min | 8% B3 |

**Table S2.** PA-011 polypeptide gradient elution procedure

| **Time** | **Velocity of flow** |
| --- | --- |
| 0-7 min | 8% B |
| 7-55 min | 12% B |
| 55-65 min | 30% B |
| 65-66 min | 40% B |
| 66-80 min | 95% B |
| 80 min | 95% B |

**Table S3.** Elution gradient condition

| **Time（Min）** | **B%** |
| --- | --- |
| 0 | 5% |
| 1 | 5% |
| 7 | 95% |
| 8 | 95% |
| 8.1 | 5% |
| 12 | 5% |

**Table S4.** The top 18 small-molecule compounds with the highest correlation.

| **Number** | **Name** | **ID** | **Molecular Formula** | **Structures** |
| --- | --- | --- | --- | --- |
| 1 | (R)-Methysticin | PA3 | C15H14O5 | 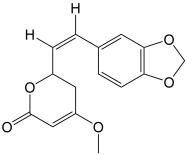 |
| 2 | 2-Phenylacetamide | PA14 | C8H9NO | 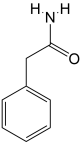 |
| 3 | 3-Hydroxyflavone | PA23 | [C15H10O3](https://pubchem.ncbi.nlm.nih.gov/#query=C15H10O3) | 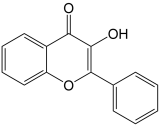 |
| 4 | 3-Methylindole | PA28 | C9H9N | 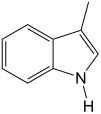 |
| 5 | Acetylcholine | PA50 | C7H16NO2+ | 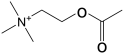 |
| 6 | Amphetamine | PA54 | C9H13N | 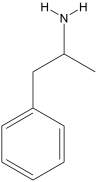 |
| 7 | Dethiobiotin | PA68 | [C10H18N2O3](https://pubchem.ncbi.nlm.nih.gov/#query=C10H18N2O3) | 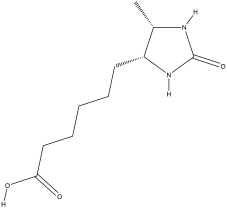 |
| 8 | Exemestane | PA76 | [C20H24O2](https://pubchem.ncbi.nlm.nih.gov/#query=C20H24O2) | 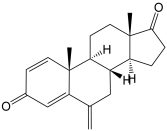 |
| 9 | Ketoprofen | PA88 | [C16H14O3](https://pubchem.ncbi.nlm.nih.gov/#query=C16H14O3) | 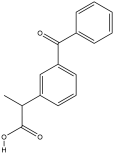 |
| 10 | Levetiracetam | PA95 | [C8H14N2O2](https://pubchem.ncbi.nlm.nih.gov/#query=C8H14N2O2) | 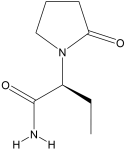 |
| 11 | L-Tryptophan | PA101 | [C11H12N2O2](https://pubchem.ncbi.nlm.nih.gov/#query=C11H12N2O2) | 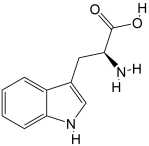 |
| 12 | N-Acetylhistamine | PA113 | [C7H11N3O](https://pubchem.ncbi.nlm.nih.gov/#query=C7H11N3O) | 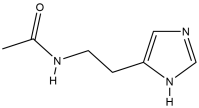 |
| 13 | N-Acetylleucine | PA115 | [C8H15NO3](https://pubchem.ncbi.nlm.nih.gov/#query=C8H15NO3) | 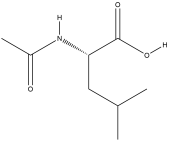 |
| 14 | N-Acetyl-L-phenylalanine | PA116 | [C11H13NO3](https://pubchem.ncbi.nlm.nih.gov/#query=C11H13NO3) | 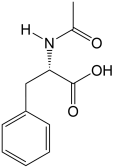 |
| 15 | Phenylethylamine | PA128 | C8H11N | 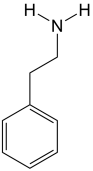 |
| 16 | trans-Isoasarone | PA149 | [C12H16O3](https://pubchem.ncbi.nlm.nih.gov/#query=C12H16O3) | 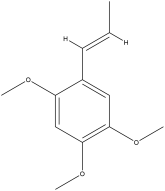 |
| 17 | Tryptophanamide | PA151 | [C11H13N3O](https://pubchem.ncbi.nlm.nih.gov/#query=C11H13N3O) | 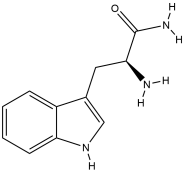 |
| 18 | Tryptophanol | PA152 | [C11H14N2O](https://pubchem.ncbi.nlm.nih.gov/#query=C11H14N2O) | 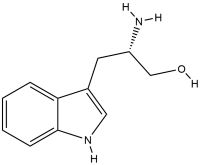 |

**Table S5.** Polypeptides used in molecular docking.

| **Number** | **Peptide** | **ID** | **Charge** |
| --- | --- | --- | --- |
| 1 | FQQRPQPQPQPQPQ | peptide13 | 1 |
| 2 | GGGAGGGAGGFGGGAGGGYR | peptide18 | 1 |
| 3 | FYGVVRAP | peptide70 | 1 |
| 4 | TPFYLR | Peptide76 | 1 |
| 5 | NFGGGSGGFGGRSP | peptide88 | 1 |
| 6 | PLNKTP | peptide98 | 1 |
| 7 | AGGGFGGGSGGFGGRSP | Peptide121 | 1 |
| 8 | AAKAAHF | peptide125 | 1.5 |
| 9 | YTKYPVYPK | peptide128 | 2 |
| 10 | RYPYAPR | peptide129 | 2 |
